# A meta-analysis of neuroimaging evidence for acupuncture-mediated modulation of altered central pain processing in patients with chronic pain

**DOI:** 10.3389/fneur.2026.1809628

**Published:** 2026-05-01

**Authors:** Xin Ma, XingXin Wang, WenHui Zhang, YuanXiang Liu, Jiguo Yang, Zhen Wang

**Affiliations:** 1 Institute of Acupuncture and Moxibustion, Shandong University of Traditional Chinese Medicine, Jinan, Shandong, China; 2 College of Acupuncture and Tuina, Shandong University of Traditional Chinese Medicine, Jinan, Shandong, China; 3 First Clinical Medical College, Shandong University of Traditional Chinese Medicine, Jinan, Shandong, China

**Keywords:** acupuncture, altered central pain processing, analgesia, brain network, chronic pain, functional magnetic resonance imaging, meta-analysis, neuroimaging

## Abstract

**Objective:**

Chronic pain, a major global public health burden, is primarily driven by altered central pain processing, which conventional treatments rarely target directly. This systematic review and meta-analysis synthesized RCT evidence to quantify acupuncture’s modulatory effects on brain networks associated with altered central pain processing, validate its clinical efficacy/safety, and explore brain network-clinical outcome associations.

**Methods:**

Comprehensive searches of English/Chinese databases (2016–2025) identified RCTs of acupuncture for chronic pain with neuroimaging. Two researchers independently performed study selection, data extraction, and bias assessment. Meta-analysis used RevMan 5.4; heterogeneity was evaluated via I^2^/Q test, with correlation analysis and GRADE evidence quality assessment.

**Results:**

Seventeen high-quality RCTs comprising 750 patients, with osteoarticular pain and migraine as main subtypes, were included. Acupuncture significantly improved neuroimaging indicators in the anterior cingulate cortex (ACC) and insula (MD = 0.27, *p* < 0.00001), primary somatosensory cortex (S1) and thalamus (MD = 0.30, *p* < 0.00001), and default mode network (DMN) (MD = 0.29, *p* < 0.00001). Clinically, acupuncture reduced Visual Analogue Scale (VAS) scores (MD = -2.31, *p* < 0.00001) and increased pain relief rate (OR = 4.30, *p* < 0.00001), with only mild adverse events reported. Osteoarticular pain demonstrated more pronounced efficacy. No significant publication bias was detected. The GRADE assessment rated the evidence for pain relief rate as high.

**Conclusion:**

Acupuncture exerts dual effects by alleviating clinical pain - exceeding the minimal clinically important difference (MCID) for VAS - and modulating brain networks implicated in altered central pain processing. It is a safe and valuable non-pharmacological intervention, with standardized protocols and subtype-specific application recommended. However, the evidence is constrained by a limited number of studies, heterogeneity in pain subtypes and neuroimaging modalities, and short follow-up durations. Larger RCTs and multimodal neuroimaging studies are needed for further validation.

**Systematic review registration:**

Registered in PROSPERO (CRD420261290299); URL: https://www.crd.york.ac.uk/prospero/.

## 1 Introduction

Chronic pain affects 20 to 30% of the global population, serving as a leading cause of disability and reduced quality of life while imposing a substantial socioeconomic burden worldwide (1). In addition to damage to peripheral tissues, altered central pain processing in spinal and supraspinal neurons is currently a key pathological mechanism of persistent pain (2–4). This neuroplastic transformation alters brain networks that regulate pain like the anterior cingulate cortex (ACC) and the insula, which are responsible for maintaining the emotional dimension of pain (5, 6). The primary somatosensory cortex (S1) and the thalamus, responsible for sorting and integrating raw sensory responses (7, 8). And the default mode network (DMN), the hub of “self-referential thinking,” which keeps us preoccupied of a lingering pain sensation (9). However, current first-line treatments - such as opioids and nonsteroidal anti-inflammatory drugs - rarely target altered central pain processing directly and are often limited by adverse effects or the development of tolerance (10, 11).

Acupuncture, a time-honored therapy rooted in traditional Chinese medicine, has been widely integrated into chronic pain management globally. It is not only clinically prevalent but also recommended in clinical practice guidelines (12, 13). While clinical trials have shown its pain-relieving effects, the neurobiological mechanisms through which acupuncture counteracts altered central pain processing are still not completely understood. Neuroimaging techniques, particularly resting-state functional magnetic resonance imaging (rs-fMRI), provide a non-invasive approach to characterizing alterations in brain network activity (14, 15). Yet existing research remains highly fragmented in terms of sample size, methodological design and outcome measurement, which has hindered the formation of a consistent consensus on the central regulatory mechanisms of acupuncture.

To address this research gap, the present systematic review and meta-analysis synthesizes data from randomized controlled trials. Its objectives are to quantify acupuncture’ s effects on neuroimaging biomarkers associated with altered central pain processing, including functional connectivity and regional homogeneity; validate its clinical analgesic efficacy and safety profile; and explore associations between brain network modulation and clinical outcomes. By integrating evidence across the neurobiological and clinical domains, this study aims to elucidate the mechanistic underpinnings of acupuncture in chronic pain management and provide evidence-based support for its application as a targeted intervention for altered central pain processing.

## 2 Methods

This systematic review and meta-analysis centers on altered central pain processing, the core pathological mechanism of chronic pain, to quantify acupuncture’s regulatory effects on neuroimaging biomarkers associated with altered central pain processing, verify its clinical analgesic efficacy and safety for chronic pain treatment, and explore the associations between acupuncture-mediated brain network modulation and clinical outcomes. The study protocol was registered with the PROSPERO International Prospective Register of Systematic Reviews (CRD420261290299).

### 2.1 Inclusion and exclusion criteria

The inclusion and exclusion criteria for this study were established in accordance with the PICO (Participants, Intervention, Comparator, Outcomes) framework. For participants, patients with a confirmed diagnosis of chronic pain were included, provided they met the universal diagnostic criteria for chronic pain with a disease duration of at least 12 weeks, a baseline Visual Analogue Scale (VAS) score of 4 or higher, an equivalent Numerical Rating Scale (NRS) score of 4 or higher, a McGill Pain Questionnaire (MPQ) score of 3 or higher, or a corresponding score on the Brief Pain Inventory (BPI). Eligible participants were aged 18 to 65 years and had no contraindications to magnetic resonance imaging (MRI) or positron emission tomography (PET) scans, such as metallic implants, claustrophobia, pregnancy, or hepatic and renal insufficiency. Patients with acute pain, cancer-related pain, postoperative pain, or psychogenic pain were excluded, as were those with a baseline VAS or equivalent NRS score below the aforementioned thresholds, individuals younger than 18 or older than 65 years, and patients with severe cardiovascular and cerebrovascular diseases, psychiatric disorders including depression and schizophrenia, or coagulation dysfunction. Also excluded were pregnant or lactating women, and those unable to cooperate with neuroimaging examinations or acupuncture interventions.

For interventions, acupuncture treatment was the primary eligible intervention, including manual acupuncture as the main modality, electroacupuncture, contralateral acupuncture, expectancy-enhanced acupuncture, and laser acupuncture (LA). For laser acupuncture, eligible studies must report core parameters in line with low-level laser therapy (LLLT) standards, including wavelength (typically 600–1,000 nm), output power (1–500 mW), energy density (ED, 0–30 J/cm^2^), irradiation time, and probe size, with acupoint selection conforming to WHO standard localization (16). For all acupuncture modalities, eligible studies needed to report clear intervention parameters: manual/electroacupuncture required needle retention time of 20 to 30 min, electroacupuncture stimulation frequency of 2 Hz, a treatment course of 1 to 16 sessions, and a total treatment cycle of 1 to 8 weeks; laser acupuncture required consistent parameter reporting as specified above. Acupuncture could be combined with routine nursing care including health education and lifestyle guidance, but not with other analgesic interventions, and all practitioners (including LA operators) were required to have at least 3 years of clinical practice qualification. Studies involving non-acupuncture interventions were excluded, such as acupressure, moxibustion, cupping therapy, acupoint injection, and oral or topical Chinese herbal medicine. Also excluded were studies with unclear intervention parameters (e.g., unreported needle retention time, electroacupuncture frequency, treatment course, or LA energy density/probe size) and those combining acupuncture with other analgesic interventions.

For comparators, eligible control interventions included sham acupuncture, conventional treatment, and blank control. Sham acupuncture modalities comprised shallow needling at non-acupoints, stimulation with blunt-tip needles, Streitberger sham needles, placebo needles, Park sham devices, sham laser acupuncture (no actual laser energy output, consistent with verum LA in probe size and irradiation duration), and phantom acupuncture. Conventional treatment included oral celecoxib, physical therapy, rehabilitation training, and treatment as usual. Blank control referred to waitlist control and routine nursing care without any analgesic intervention. Studies without a clear control intervention or those using only pre-post self-control designs were excluded.

For outcomes, core outcomes were neuroimaging indices, including rs-fMRI metrics with analytical methods such as fractional amplitude of low-frequency fluctuation (fALFF), regional homogeneity (ReHo), resting-state functional connectivity (rsFC), and degree centrality (DC). These were assessed in brain regions including the DMN, sensorimotor network, thalamus, ACC, and dorsolateral prefrontal cortex (DLPFC). Structural MRI indices, including voxel-based morphometry (VBM) and fractional anisotropy (FA), PET indices such as standardized uptake value (SUV) and standardized uptake value ratio (SUVR), and proton magnetic resonance spectroscopy (^1^H-MRS) indices including gamma-aminobutyric acid (GABA) concentration were also included as core outcomes. Secondary outcomes consisted of clinical indices and safety indices. Clinical indices included changes in VAS/NRS scores, pressure pain threshold (PPT), pain relief rate, and functional and psychological scales such as the Oswestry Disability Index (ODI), Western Ontario and McMaster Universities Osteoarthritis Index (WOMAC), MPQ, and Headache Impact Test-6 (HIT-6). Safety indices were the incidence of adverse events. Associative outcomes, defined as the correlation coefficient “r” between neuroimaging indices and clinical outcomes, were also included. Studies without core neuroimaging indices, those reporting only a single clinical index with no neuroimaging data or associative analysis results, and studies with non-standard outcome measurement methods, such as unreported time points for VAS score assessment, were excluded. For study design, only randomized controlled trials were included, regardless of blinding status or multicenter design. Non-RCT study designs were excluded, including cohort studies, case–control studies, cross-sectional studies and case reports. Also excluded were review articles, animal experiments and basic laboratory studies.

### 2.2 Literature search strategy

A systematic literature search was conducted across English databases (PubMed, EMBASE, the Cochrane Library, Web of Science, Scopus), Chinese databases (China National Knowledge Infrastructure, Wanfang Data Knowledge Service Platform, VIP Chinese Science and Technology Journal Database, China Biological Medicine Database) and grey literature sources, including clinical trial registration platforms (ClinicalTrials.gov, Chinese Clinical Trial Registry), conference proceedings and dissertations. The search timeframe was set from January 1, 2016 to December 31, 2025. A combined search strategy of subject headings and free text terms was adopted. Taking PubMed as an example, the core search string was constructed as follows: (“acupuncture”[Mesh] OR “acupuncture therapy”[Mesh] OR acupuncture[tiab] OR electroacupuncture[tiab] OR needling[tiab]) AND (“chronic pain”[Mesh] OR chronic pain[tiab] OR “brain plasticity”[tiab]) AND (“neuroimaging”[Mesh] OR “magnetic resonance imaging”[Mesh] OR “positron emission tomography”[Mesh] OR fMRI[tiab] OR “functional magnetic resonance imaging”[tiab] OR PET[tiab] OR “positron emission tomography”[tiab] OR “default mode network”[tiab] OR DMN[tiab] OR “sensorimotor network”[tiab] OR “anterior cingulate cortex”[tiab] OR ACC[tiab] OR insula[tiab] OR “somatosensory cortex”[tiab] OR thalamus[tiab]) AND (“randomized controlled trial”[Publication Type] OR “randomized controlled trial”[tiab] OR RCT[tiab]). Equivalent search strategies were adapted for other databases according to their respective syntax requirements.

### 2.3 Study selection and data extraction

The study selection process was carried out as follows: duplicate records were first removed through automatic de-duplication via EndNote X9 software combined with manual verification. Subsequently, two independent researchers screened titles and abstracts to exclude studies that clearly failed to meet the inclusion and exclusion criteria, after which full texts were assessed one by one against the same criteria to confirm the final included studies. In case of discrepancies during the screening process, a third independent researcher was consulted to reach a consensus, with a target discrepancy rate of no more than 5%.

A standardized data extraction form was developed using Excel 2021 for data extraction, which was independently conducted by two researchers and cross-checked for consistency. The extracted information included the basic study characteristics (first author, year of publication, country or region, clinical trial registration number, funding source, etc.), participant demographics (pain subtype, sample size, age, gender ratio, disease duration, baseline VAS, NRS and MPQ scores, etc.), intervention and control details (type of acupuncture, acupoint selection, intervention parameters, deqi induction, type and implementation of control intervention, treatment course, etc.), outcome data (effect sizes, 95% confidence intervals, *p* values and sample sizes for neuroimaging, clinical and associative outcomes, etc.), and information relevant to risk of bias assessment (method of random sequence generation, allocation concealment, blinding implementation, loss to follow-up rate, completeness of outcome data, etc.).

### 2.4 Risk of bias assessment

The Cochrane Risk of Bias Tool version 2.0 (RoB 2.0) was used to assess the risk of bias across all included randomized controlled trials. The assessment covered multiple domains: random sequence generation, allocation concealment, blinding implementation for participants, therapists and outcome assessors, completeness of outcome data, selective reporting, and other potential sources of bias such as baseline imbalance and conflicts of interest. Each domain was rated into three tiers: low risk of bias, high risk of bias, and some concerns. The assessment was independently conducted by two researchers, and any discrepancies arising during the process were resolved through consultation with a third independent researcher.

### 2.5 Statistical analysis methods

Meta-analysis was performed using RevMan 5.4 software with a significance level of *α* = 0.05. Heterogeneity was assessed using the *I*^2^ statistic combined with the *Q* test: a result of *I*^2^ < 50% and *p* > 0.10 indicated good homogeneity, and the fixed-effects model was thus applied; in cases where *I*^2^ ≥ 50% or *p* ≤ 0.10 suggested the presence of heterogeneity, the random-effects model was adopted, with subgroup and sensitivity analyses conducted to explore the sources of heterogeneity. Spearman’s rank correlation analysis was conducted to explore the bivariate associations among imaging index changes, clinical pain changes and imaging-clinical correlation coefficients using R software (Version 4.4.2, R Foundation for Statistical Computing, Vienna, Austria). The ‘cor.test’ function with the ‘method = “spearman” argument was employed to calculate Spearman’s rank correlation coefficient (r) and corresponding two-tailed *p*-values, with a significance level set at *α* = 0.05.

For effect size pooling, the standardized mean difference (SMD) or mean difference (MD) was used as the effect size for continuous variables, including *β* values, *Z* values, ReHo values, fALFF values, SUV values, changes in VAS/NRS scores, PPT, GMV and FA values. The odds ratio (OR) was used as the effect size for dichotomous variables, namely pain relief rates and adverse event rates, with 95% confidence intervals calculated for all effect sizes. Funnel plot analysis was conducted to detect publication bias, and the trim-and-fill method was applied for correction if publication bias was identified.

Subgroup analyses were stratified by pain subtype, control intervention type (sham acupuncture, conventional treatment, blank control) and acupuncture intervention parameters (manual acupuncture vs. electroacupuncture, needle retention time < 20 min vs. ≥ 20 min, treatment course < 6 weeks vs. ≥ 6 weeks, and deqi induction applied or not). The GRADE approach was used to assess the quality of evidence, which involved evaluations across five domains: risk of bias, inconsistency (heterogeneity), indirectness, imprecision and publication bias. The quality of evidence was categorized into four levels: high, moderate, low and very low.

## 3 Results

### 3.1 Study identification and selection outcomes

**Figure 1:**
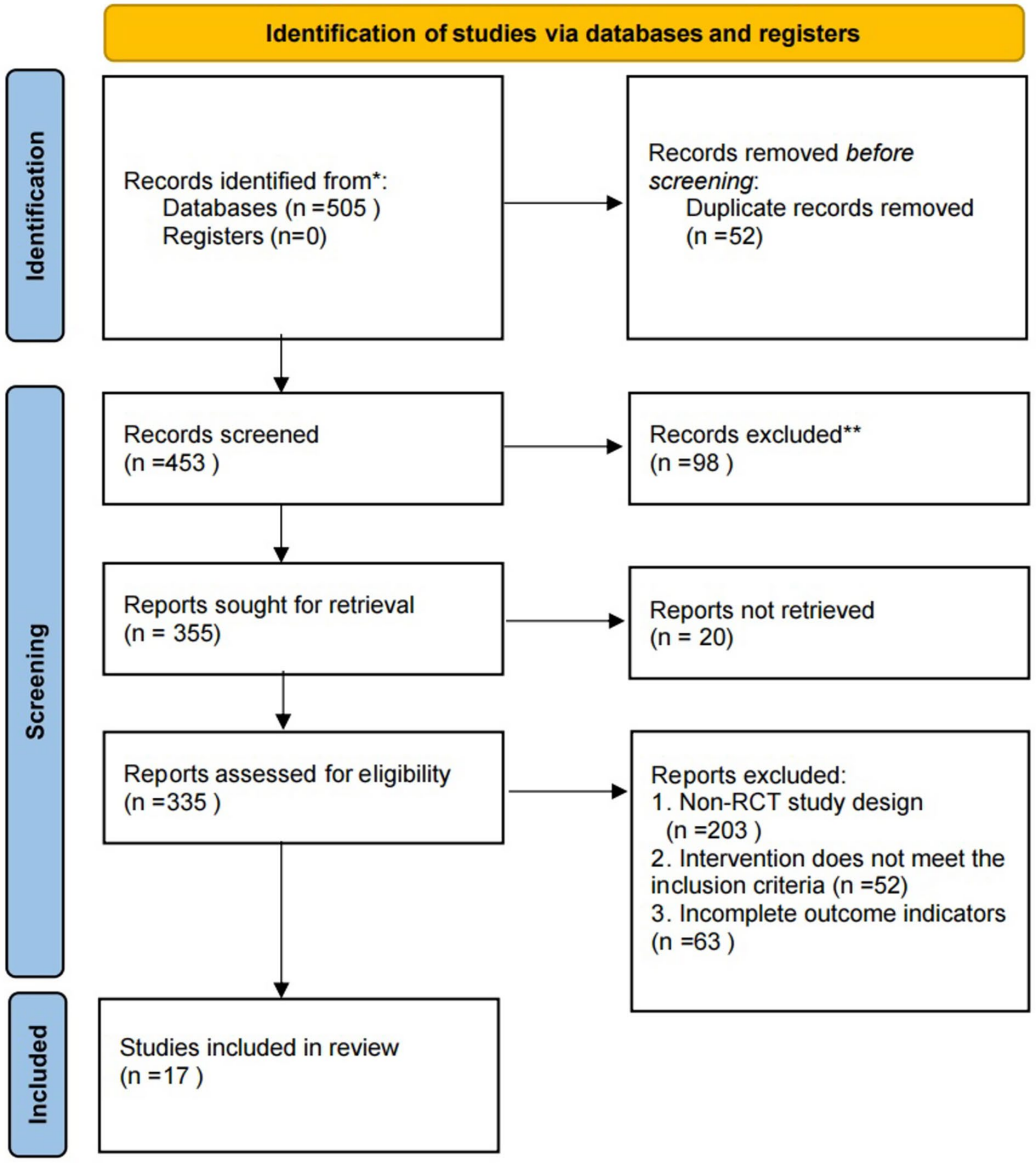
Flow chart of literature screening.

A preliminary search across electronic databases and clinical trial registration platforms yielded a total of 505 records. After the removal of 52 duplicate records via automatic de-duplication in EndNote X9 software, 453 studies proceeded to the title and abstract screening stage. A total of 98 studies were excluded at this stage for failing to meet the inclusion and exclusion criteria, leaving 355 studies for full-text retrieval. Of these, 20 studies were excluded due to unobtainable full texts, with the remaining 335 studies undergoing full-text eligibility assessment. Following full-text evaluation, 318 studies were excluded for various ineligibilities, including 203 non-randomized controlled trials, 52 studies with ineligible interventions, and 63 studies with incomplete outcome measures. Ultimately, 17 randomized controlled trials were included in the final analysis, categorized by chronic pain subtype: 4 studies focused on osteoarticular pain, 3 on migraine, 3 on chronic low back pain (cLBP), and 7 on other subtypes (including fibromyalgia and joint pain). The detailed study selection process is illustrated in Figure 1.

### 3.2 Baseline characteristics of included studies

A total of 17 studies (17–33) were included in this meta-analysis (Table 1), with articular pain and migraine constituting the primary subtypes of chronic pain investigated. The sample size of individual studies ranged from 20 to 149 participants, and manual acupuncture was the predominant intervention in the experimental groups. Sham acupuncture was used as the control in 13 studies, with waitlist and routine care serving as alternative control interventions in the remaining trials.

**Table 1:** Baseline characteristics of included studies.

Author, year	Pain subtype	Sample size (Intervention/Control Group)	Baseline pain score (VAS/NRS, score)	Acupuncture intervention	Control intervention	Primary outcome measures
XiaoYa Wei, 2024	Chronic Sciatica (Neuropathic Pain)	Acupuncture: 25/Sham Acupuncture: 25 (Completed: 50 cases)	Acupuncture: 50.00 (45.00–71.50) mm; Sham Acupuncture: 50.00 (45.00–58.00) mm	Manual acupuncture; BL25, BL26, etc.; Retention time: 30 min; 10 sessions over 4 weeks; Licensed acupuncturist (≥3 years of experience)	Sham acupuncture (non-acupoint blunt needle, no skin penetration)	1. Imaging: rs-fMRI-fALFF (right SPL, PoCG); 2. Clinical: Leg pain VAS, ODI, SFBI
Xu Wang, 2023	Knee Osteoarthritis (KOA)-Related Pain	Acupuncture: 27 / Sham Acupuncture: 25(Completed: 52 cases)	Baseline NRS not reported with specific values	Standardized manual acupuncture; Acupoints not specified; 12 sessions over 4 weeks (≥10 sessions)	Sham acupuncture, blank control (waitlist); Operation not specified	1. Imaging: rs-fMRI-fALFF, cortical thickness, brain network small-world property; 2. Clinical: NRS, WOMAC
Chong Li, 2023	Migraine	Acupuncture: 12 / Sham Acupuncture: 13 / Blank Control: 13 (Completed: 38 cases)	Acupuncture: 7.0 ± 1.4; Sham Acupuncture: 7.0 ± 1.6; Blank Control: 6.5 ± 1.3	Manual acupuncture; GB20, LR3, EX-HN5, etc.; Lifting-thrusting and twisting manipulation; Retention time: 30 min; 10 sessions over 2 courses	Sham acupuncture (non-acupoint shallow insertion 4 mm), blank control	1. Imaging: rs-fMRI-fALFF/ReHo (lingual gyrus, temporal lobe, etc.); 2. Clinical: VAS, attack frequency, PSQI
Lu Liu, 2022	Migraine	True Acupuncture: 20/Sham Acupuncture: 20 (Completed: 40 cases)	True Acupuncture: 7.60 ± 1.50; Sham Acupuncture: 7.50 ± 1.54	Manual acupuncture; GB20, GV16, GV20, etc.; Lifting-thrusting and twisting (90°-180°); Retention time: 30 min (manipulation every 10 min); 12 sessions over 4 weeks	Sham acupuncture (non-acupoint shallow insertion 10-15 mm)	1. Imaging: rs-fMRI-rsFC (AMYG, MCC, etc.); 2. Clinical: VAS, MMDs, HIT-6
Jun Zhou, 2023	KOA-Related Pain	True Acupuncture: 28/Sham Acupuncture: 32/Celecoxib: 29/Placebo: 30/Waitlist: 30 (Completed: 149 cases)	True Acupuncture: 4.00 (3.00–5.00); Sham Acupuncture: 4.00 (3.00–5.00)	Manual acupuncture; GB34, SP9, ST35, etc.; Deqi induction; Retention time: 30 min; 10 sessions over 2 weeks	Sham acupuncture (non-acupoint, insertion 0.5–1.5 cun), celecoxib, placebo, waitlist	1. Imaging: rs-fMRI-rsFC (vlPAG, DLPFC, etc.); 2. Clinical: VAS, SF-MPQ, WOMAC
Ishtiaq Mawla, 2019	Fibromyalgia (FM)	Electroacupuncture (EA): 36/Sham Laser Acupuncture: 34 (Completed: 70 cases)	BPI Severity baseline not reported (1.14-point reduction in EA group after intervention)	Electroacupuncture; LI4-LI11, GB34-SP6, etc.; Low frequency: 2 Hz; Retention time: 25 min; 8 sessions over 4 weeks; Licensed acupuncturist	Sham laser acupuncture (no actual laser stimulation)	1. Imaging: rs-fMRI-rsFC, ^1^H-MRS-GABA+ (S1leg, aINS, etc.); 2. Clinical: BPI Severity
Jian Kong, 2018	KOA-Related Pain	Acupuncture: 19/Standard Acupuncture: 18/Usual Care: 17 (Completed: 54 cases, analyzed: 46 cases)	KOOS Pain Subscale: Enhanced Expectation: 57.7 ± 13.9; Standard: 68.6 ± 13.2; Usual Care: 63.7 ± 15.6	Manual acupuncture; ST35, EX-LE5, GB34, etc.; Twisting (120 rotations/min); Retention time: 20 min; 10 sessions over 8 weeks	Standard acupuncture (no expectation enhancement), usual care (TAU)	1. Imaging: rs-fMRI-rsFC, ^1^H-MRS-GABA+ (NAc, MPFC, etc.); 2. Clinical: KOOS Pain Subscale
Chaorong Xie, 2025	Migraine	True Acupuncture: 32/Sham Acupuncture: 30 (Clinical: 62 cases, Imaging: 56 cases)	True Acupuncture: 5.20 ± 1.52; Sham Acupuncture: 5.75 ± 1.20	Manual acupuncture; GV20, GB20, GB8, etc.; Deqi induction; Retention time: 30 min; 12 sessions over 4 weeks	Sham acupuncture (Park sham device, non-acupoint blunt needle)	1. Imaging: rs-fMRI-ALFF/fALFF/ReHo/DC (DMN, SMN, etc.); 2. Clinical: VAS, attack frequency/duration
Hyungjun Kim, 2020	Chronic Low Back Pain (cLBP)	True Acupuncture: 18/Sham Acupuncture: 18/Sham Laser: 19/Usual Care: 23 (Completed: 70 cases)	Pain interference score: 5.4 ± 2.1 (VAS-equivalent)	Manual acupuncture; GV3, BL23, BL40, etc.; Twisting (2 Hz); Retention time: 20 min; 6 sessions over 4 weeks; Licensed acupuncturist (≥3 years)	Sham acupuncture (blunt retractable needle), sham laser, usual care (TAU)	1. Imaging: Structural MRI-VBM/FA (S1-back, etc.); 2. Clinical: Pain interference score, 2PDT
Xiao Wang, 2023	Chronic neck pain (CNP)	True Acupuncture: 66 / Sham Acupuncture: 33 (Clinical: 97 cases, Imaging: 92 cases)	True Acupuncture: 58.14 ± 13.2 mm; Sham Acupuncture: 54.78 ± 13.3 mm	Manual acupuncture; SJ15, SI14, BL11, etc.; Lifting-thrusting and twisting for Deqi; Retention time: 30 min; 12 sessions over 4 weeks; Licensed acupuncturist (≥5 years)	Sham acupuncture (non-acupoint shallow insertion 2 mm)	1. Imaging: rs-fMRI-rsFC (DR, MR, thalamus, etc.); 2. Clinical: VAS, NDI, SAS
Jin Xu, 2022	Primary dysmenorrhea (PDM)	True acupuncture: 14/Sham acupuncture: 15 (Completed: 29 cases)	True acupuncture: 6.07 ± 1.07; Sham Acupuncture: 6.00 ± 1.20	Manual acupuncture; SP6; Lifting-thrusting and twisting for Deqi; Retention time: 30 min; 3 menstrual cycles (≥4 weeks); Licensed acupuncturist (≥3 years)	Sham acupuncture (shallow insertion at midpoint of SP6 and GB39)	1. Imaging: PET-SUVR (DMN, SMN, etc.); 2. Clinical: VAS, SAS, SDS
ChaoQun Yan, 2020	Chronic shoulder pain (CSP)	Contralateral acupuncture: 10/Ipsilateral Acupuncture: 10 (Completed: 20 cases)	VAS-equivalent ≥50 mm (5 points)	Manual acupuncture; ST38 (contralateral/ipsilateral); Lifting-thrusting and twisting for Deqi; Retention time: 20 min (active shoulder joint movement); Single session	Ipsilateral acupoint acupuncture (ST38 on painful side)	1. Imaging: rs-fMRI-DC (ACC, S1, cerebellum, etc.); 2. Clinical: VAS, CMS score
XiaoYa Wei, 2025	KOA-related pain	Acupuncture: 27/Sham acupuncture: 25/Waitlist: 22 (Completed: 74 cases)	Acupuncture: 6.00 (5.00–7.00); Sham Acupuncture: 5.00 (4.50–7.00); Waitlist: 5.00 (4.00–6.00)	Manual acupuncture; ST35, EX-LE4, LR8, etc. + Ashi points; Lifting-thrusting and twisting for Deqi; Retention time: 30 min; 12 sessions over 4 weeks; Licensed acupuncturist (≥5 years)	Sham acupuncture (non-acupoint blunt needle), waitlist (blank control)	1. Imaging: rs-fMRI-ALFF/rsFC (DLPFC, thalamus, etc.); 2. Clinical: NRS, WOMAC, pain relief rate
Jeungchan Lee, 2019	Chronic low back pain (cLBP)	True acupuncture: 25/Phantom Acupuncture: 18 (Completed: 43 cases)	LBE pain model: True Acupuncture: 4.74 ± 1.00; Phantom Acupuncture: 5.54 ± 0.83	Manual acupuncture; SP13, SP11, ST36, etc.; Twisting (1 Hz); Retention time: 7 min; Single session	Phantom acupuncture (video feedback + verbal instruction, no actual insertion)	1. Imaging: rs-fMRI-rsFC (DMN, SN, SMN, etc.); 2. Clinical: LBE pain score
Yiheng Tu, 2019	Chronic low back pain (cLBP)	True acupuncture: 24/Sham Acupuncture: 26 (Completed: 50 cases)	Same as disease duration (data duplication in original article, filled as disease duration)	Manual acupuncture; GV3, BL23, BL40, etc. + Ashi points; Lifting-thrusting and twisting for Deqi; Retention time: 25 min; 6 sessions over 4 weeks; Licensed acupuncturist	Sham acupuncture (Streitberger placebo needle, non-acupoint pressure)	1. Imaging: rs-fMRI-rsFC (DMN, SN, etc.), SVR prediction model; 2. Clinical: VAS, PROMIS score
ChengHao Tu, 2021	Primary dysmenorrhea (PDM)	True acupuncture: 18/Sham Acupuncture: 16 (Clinical: 34 cases, Imaging: 27 cases)	MPQ-PPI: True Acupuncture: 3.17 ± 1.20; Sham Acupuncture: 3.25 ± 1.00 (VAS-equivalent ≥4 points)	Manual acupuncture; Bilateral SP6; Vertical insertion without additional stimulation; Retention time: 20 min; 16 sessions over 8 weeks; Licensed acupuncturist (≥10 years)	Sham acupuncture (Streitberger placebo needle, non-acupoint pressure)	1. Imaging: rs-fMRI-rsFC (PAG, dACC, etc.); 2. Clinical: MPQ-PRI/PPI
Shuai Zhang, 2018	Chronic shoulder pain (CSP)	Contralateral acupuncture: 12/Ipsilateral acupuncture: 8 (Completed: 20 cases)	Contralateral: 67.92 ± 16.16 mm; Ipsilateral: 60.63 ± 7.29 mm	Manual acupuncture; ST38 (contralateral/ipsilateral); Lifting-thrusting and twisting (180°) for Deqi; Retention time: 20 min (active shoulder joint movement); Single session	Ipsilateral acupoint acupuncture (ST38 on painful side)	1. Imaging: rs-fMRI-ReHo (ACC, thalamus, etc.); 2. Clinical: VAS, CMS score

“Completed cases” in the sample size column refers to the final sample size included in statistical analysis; the number outside the parentheses is the initial grouping sample size. For baseline pain score, median (interquartile range) was used if mean ± SD was not clearly reported in the original study. “Deqi induction” in the acupuncture intervention column refers to the production of soreness, numbness, distension, or radiation sensation through lifting-thrusting, twisting, or other manipulations. For primary outcome measures, imaging indicators specify the detection modality (rs-fMRI/Structural MRI/PET) and core brain regions, while clinical indicators prioritize pain scores (VAS/NRS/MPQ) and key functional scores.

The mean age of the study participants ranged from 24.53 to 63.00 years, and the median disease duration was 3 to 11.44 years, with baseline VAS or NRS pain scores between 4.00 and 7.60 points across all included studies. Acupuncture interventions were primarily performed at classic acupoints with an emphasis on deqi induction; the needle retention time was 20 to 30 min, the total treatment course consisted of 1 to 16 sessions, and all acupuncture procedures were administered by qualified practitioners. All studies reported both neuroimaging and clinical outcome measures: rs-fMRI was the main neuroimaging modality, with a focus on brain networks including the DMN and sensorimotor network, while the VAS and NRS pain scales were the core clinical outcome measures.

### 3.3 Risk of bias assessment results

**Figure 2:**
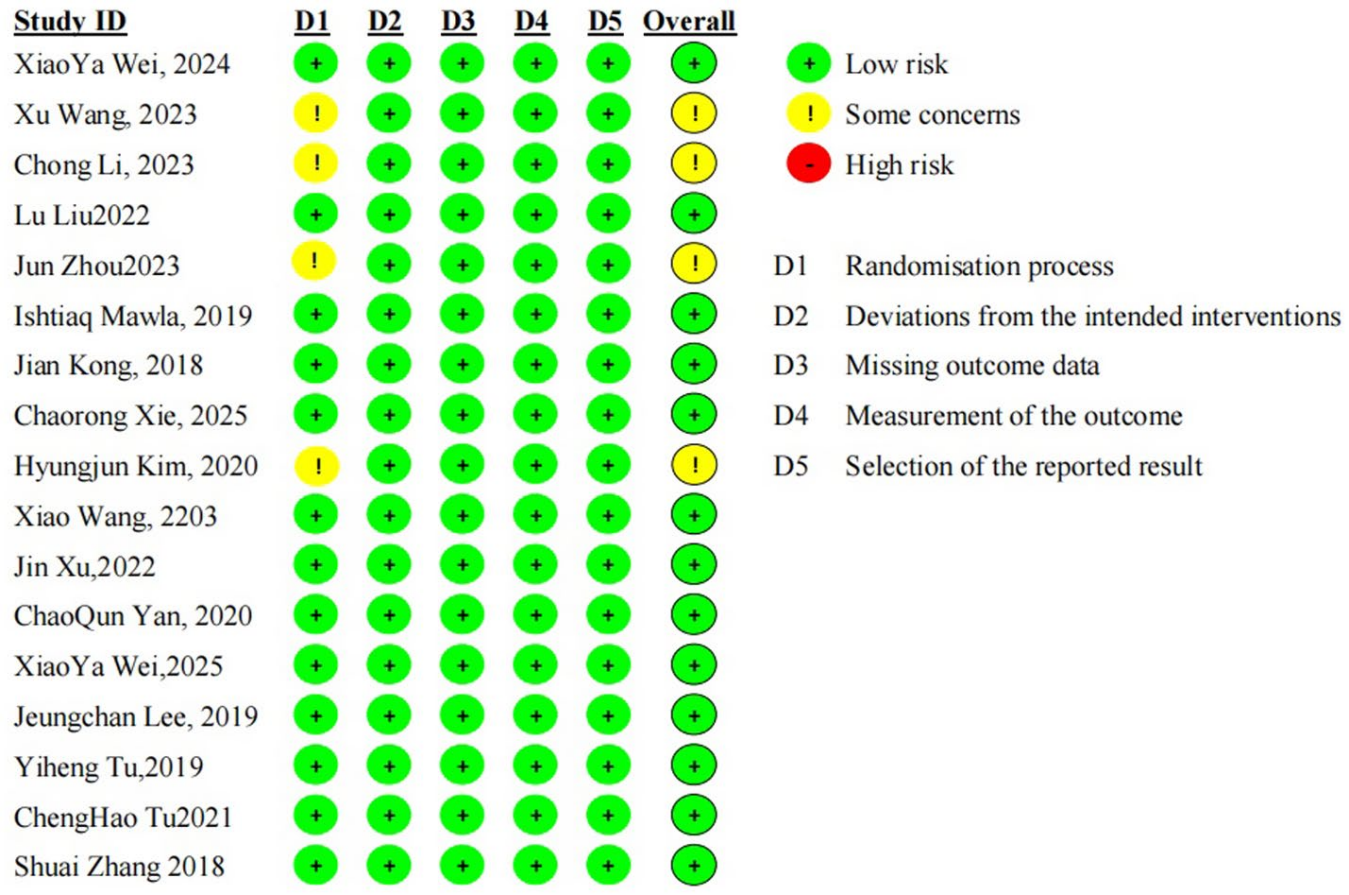
Cochrane Risk of Bias (RoB 2.0) assessment results of included studies. D1 = Randomisation process; D2 = Deviations from the intended interventions; D3 = Missing outcome data; D4 = Measurement of the outcome; D5 = Selection of the reported result; “+” indicates low risk of bias, “!” indicates some concerns.

The Cochrane Risk of Bias Tool version 2.0 (RoB 2.0) was applied to independently assess the risk of bias across the 17 included randomized controlled trials (RCTs), with evaluations conducted for five core domains: randomization process (D1), deviations from the intended interventions (D2), missing outcome data (D3), measurement of the outcome (D4), and selective reporting (D5). The results are presented in Figure 2: 13 studies (17, 18, 20, 22–24, 27–33) showed no significant risk of bias across all five domains and were thus rated as having an overall low risk of bias. The remaining four studies (7, 19, 21, 25) exhibited potential bias concerns only in the randomization process domain (D1), with a low risk of bias identified in the other four domains; these studies were consequently rated as having some concerns overall. This finding indicated insufficient reporting of information related to random sequence generation or allocation concealment in these trials, though no definitive high-risk bias factors were detected. None of the trials included in this meta-analysis were rated as having a high risk of bias, reflecting good overall quality in bias control and providing reliable evidentiary support for subsequent effect size pooling and result analysis.

### 3.4 Results of meta-analysis

#### 3.4.1 Neuroimaging indicators related to altered central pain processing

**Figure 3:**
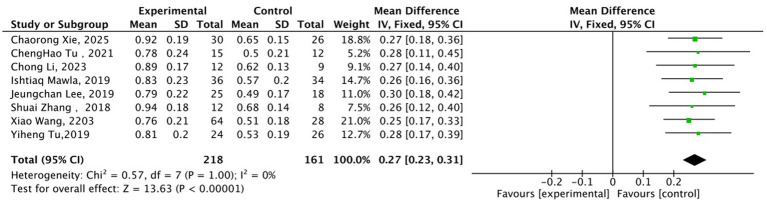
Forest plot of the comparison between acupuncture group and sham acupuncture group for pain emotion/perception regulation-related neuroimaging indicators. MD = Mean Difference; CI = Confidence Interval; fALFF = fractional Amplitude of Low-Frequency Fluctuations; ReHo = Regional Homogeneity; rsFC = resting-state Functional Connectivity.

In the dimension of pain emotion/perception regulation, a total of 8 studies (19, 22, 24, 26, 29–32) (218 cases in the acupuncture group, 161 cases in the sham acupuncture group) focusing on the ACC and insula as core target brain regions were included, with analysis indicators covering fMRI-fALFF, ReHo, and rsFC. No statistical heterogeneity was observed across studies (*I*^2^ = 0%, *p* = 1.00). Results from the fixed-effects model (Figure 3) demonstrated that the improvement magnitude of relevant imaging indicators in the acupuncture group was significantly superior to that in the sham acupuncture group (MD = 0.27, 95% CI: 0.23–0.31, Z = 13.63, *p* < 0.00001), suggesting that acupuncture can effectively regulate the pain emotion-perception circuit involving the ACC and insula.

**Figure 4:**
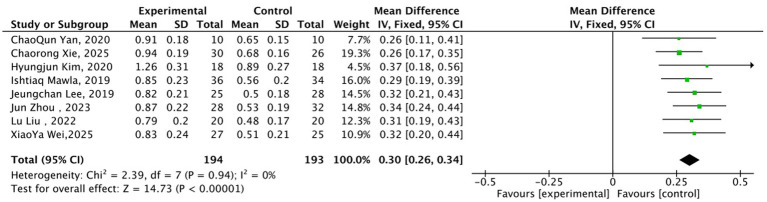
Forest plot of the comparison between acupuncture group and sham acupuncture group for sensory information integration-related neuroimaging indicators. MD = Mean Difference; CI = Confidence Interval; rsFC = resting-state Functional Connectivity; GMV = Gray Matter Volume; FA = Fractional Anisotropy.

In the dimension of sensory information integration, 8 studies (20–22, 24, 25, 28, 29, 33) (194 cases in the acupuncture group, 193 cases in the sham acupuncture group) focusing on the primary S1 and thalamus were included, involving fMRI-rsFC, gray matter volume (GMV), and FA indicators. No significant heterogeneity was detected among studies (*I*^2^ = 0%, *p* = 0.94). Under the fixed-effects model, the acupuncture group exhibited a significantly greater improvement in indicators compared to the sham acupuncture group (MD = 0.30, 95% CI: 0.26–0.34, Z = 14.73, *p* < 0.00001) (Figure 4), indicating that acupuncture can enhance the sensory information integration function of the S1 and thalamus.

**Figure 5:**
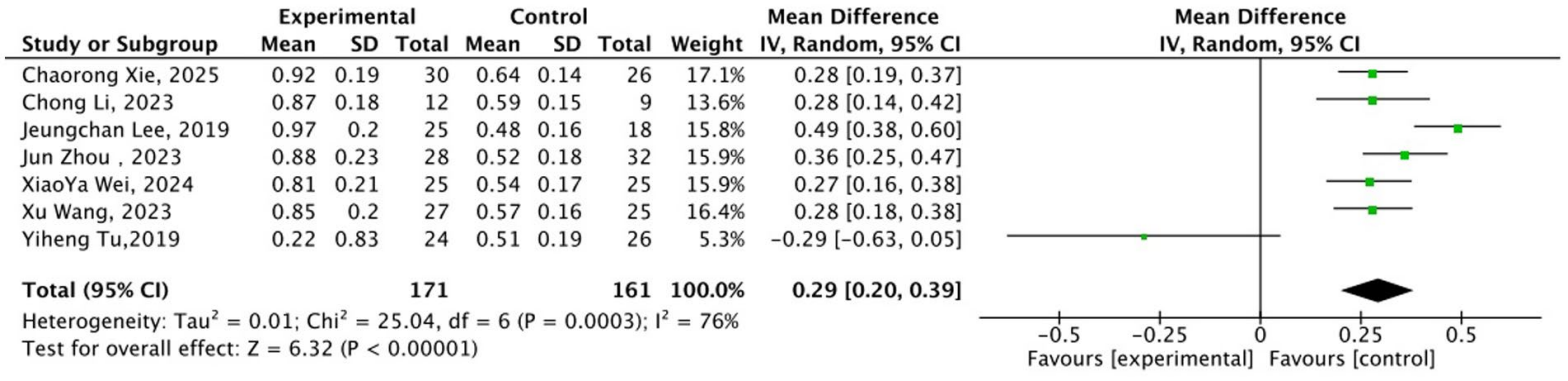
Forest plot of the comparison between acupuncture group and sham acupuncture group for default mode network (DMN) function regulation-related neuroimaging indicators. MD = Mean Difference; CI = Confidence Interval; ReHo = Regional Homogeneity; ALFF = Amplitude of Low-Frequency Fluctuations; rsFC = resting-state Functional Connectivity.

In the dimension of DMN function regulation, 7 studies (17, 19, 21, 24, 26, 29, 30) (171 cases in the acupuncture group, 161 cases in the sham acupuncture group) targeting the precuneus/posterior cingulate cortex (PCC) were included, with analysis indicators including fMRI-ReHo, ALFF, and rsFC. High heterogeneity was observed across studies (*I*^2^ = 76%, *p* = 0.0003). After analysis using the random-effects model, the acupuncture group still showed significantly better improvement in indicators than the sham acupuncture group (MD = 0.29, 95% CI: 0.20–0.39, Z = 6.32, *p* < 0.00001) (Figure 5). Subsequent subgroup analyses can further explain the source of heterogeneity, suggesting that acupuncture can correct DMN dysfunction in chronic pain states.

#### 3.4.2 Clinical outcome measures

**Figure 6:**
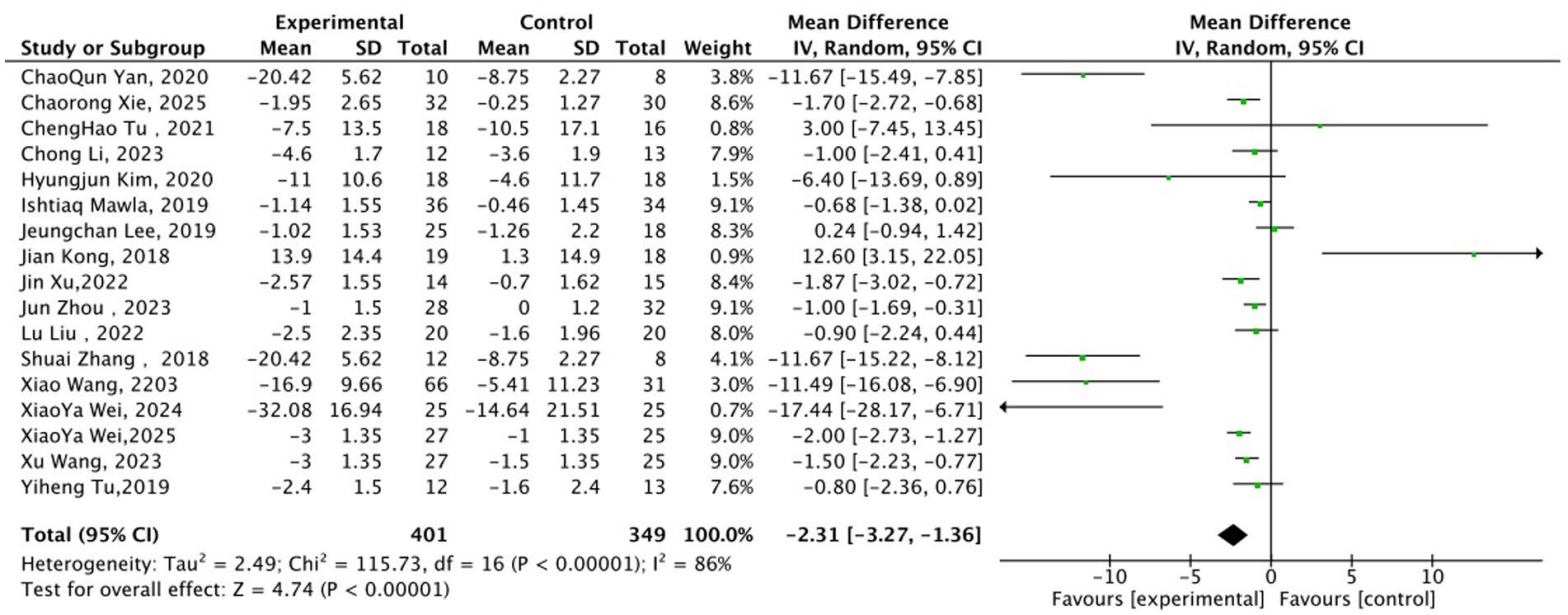
Forest plot of Visual Analogue Scale (VAS) score changes between acupuncture group and control group. MD = Mean Difference; CI = Confidence Interval; NRS = Numerical Rating Scale; MPQ = McGill Pain Questionnaire.

For the analysis of changes in Visual Analogue Scale (VAS) scores (and equivalent pain scores), results from the random-effects model of 17 studies (401 cases in the acupuncture group, 349 cases in the control group) showed that the magnitude of pain score reduction in the acupuncture group was significantly greater than that in the control group (Figure 6), with a pooled mean difference (MD) of −2.31 (95% CI: −3.27 to −1.36, Z = 4.74, *p* < 0.00001). This indicated that acupuncture exerts a definite clinical efficacy in relieving chronic pain. Heterogeneity tests revealed high statistical heterogeneity across the included studies (*I*^2^ = 86%, χ^2^ = 115.73, df = 16, *p* < 0.00001). The heterogeneity was speculated to mainly originate from differences in pain subtypes (e.g., distinct pathological mechanisms between osteoarticular pain and migraine), inconsistent metrics of outcome measures (some studies adopted Numerical Rating Scale (NRS) or McGill Pain Questionnaire (MPQ) scores instead of VAS), diversity in acupuncture manipulation techniques (variations in stimulation parameters between manual acupuncture and electroacupuncture), and differences in control types (varying placebo effects of sham acupuncture, conventional therapy and blank control).

**Figure 7:**
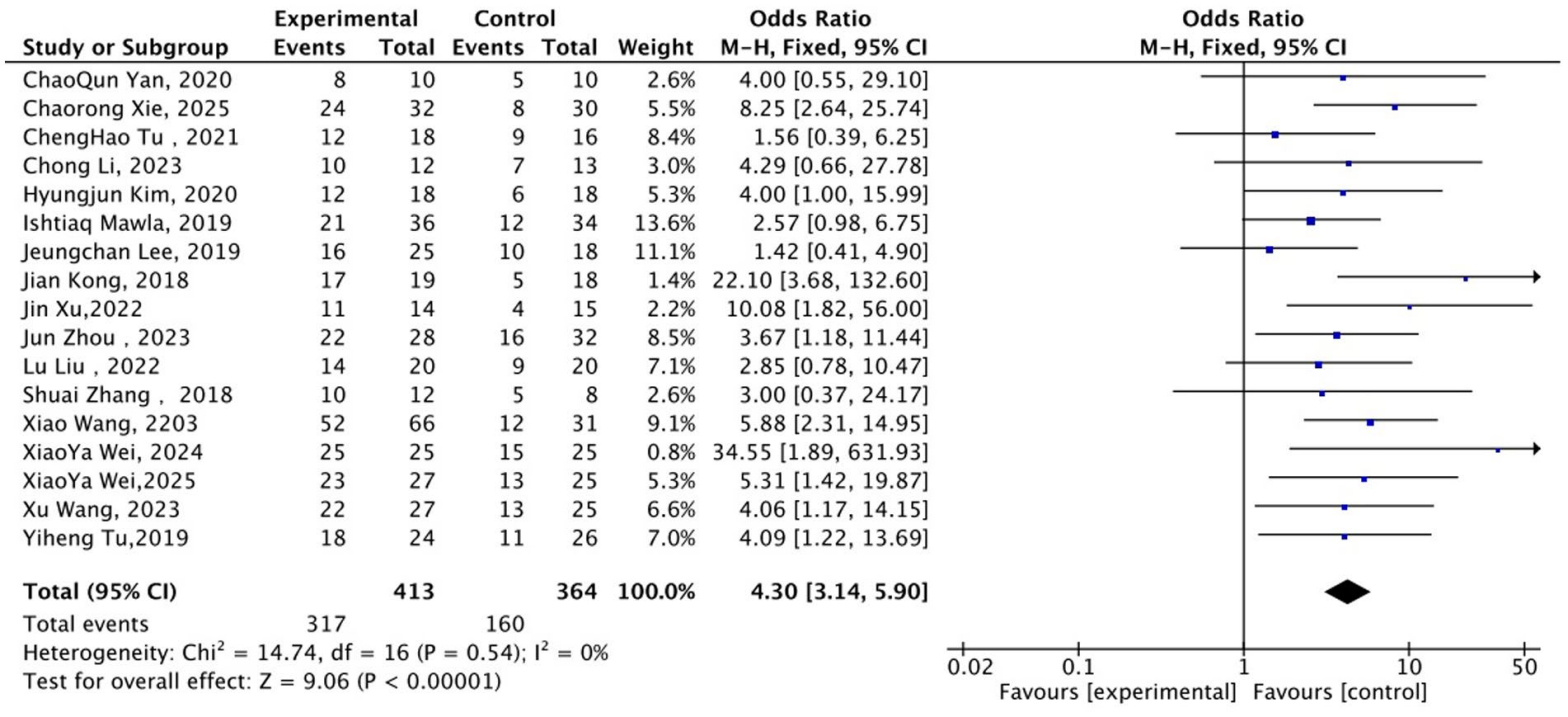
Forest plot of pain relief rate between acupuncture group and control group. OR = Odds Ratio; CI = Confidence Interval; VAS = Visual Analogue Scale; NRS = Numerical Rating Scale.

A fixed-effects model was applied for the meta-analysis of pain relief rate (defined as a reduction of ≥2 points in VAS/NRS scores or equivalent criteria). The results demonstrated that the proportion of patients achieving the clinical pain relief standard in the acupuncture group was significantly higher than that in the control group (Figure 7), with a pooled odds ratio (OR) of 4.30 (95% CI: 3.14 to 5.90, *Z* = 9.06, *p* < 0.00001). This suggested that the probability of obtaining pain relief in the acupuncture group was 4.3 times that in the control group. Heterogeneity tests showed no statistical heterogeneity across studies (χ^2^ = 14.74, df = 16, *p* = 0.54; *I*^2^ = 0%), indicating good consistency in the results of different studies. This consistency was largely attributed to the adoption of standardized acupuncture protocols and sham acupuncture-controlled designs in most included studies, which reduced the heterogeneity of intervention measures. Overall, the available evidence indicated that acupuncture can significantly improve the pain relief rate in patients with chronic pain with high consistency across study results, supporting the clinical value of acupuncture as an effective intervention for chronic pain.

In terms of safety, the overall incidence of adverse events across the 17 studies was low, all of which were mild local reactions, with no serious adverse events reported (Supplementary Table S1). Mild acupuncture-related adverse events were reported in only 6 studies (17, 21, 24, 26, 31), including ecchymosis around acupoints, subcutaneous hemorrhage and post-acupuncture discomfort, with a maximum of 4 cases in a single study. All these events resolved spontaneously within 1–2 weeks without the need for special intervention. One study reported 1 case in each group taking analgesics for non-dysmenorrhea reasons (31), and no adverse events were reported in the remaining studies. These findings suggested that acupuncture has a favorable safety profile for the treatment of chronic pain, with adverse events predominantly being self-limiting local reactions.

#### 3.4.3 Correlation analysis

Spearman’s rank correlation analysis revealed (Table 2) a strongly positive correlation between the changes in neuroimaging indicators and the imaging-clinical correlation coefficient (*r* = 0.931, *p* = 5.74 × 10^−8^ < 0.001), suggesting that the magnitude of changes in neuroimaging indicators after acupuncture intervention was highly synchronized with the strength of the imaging-clinical association. In contrast, no significant correlations were observed between the changes in neuroimaging indicators and the changes in clinical pain scores (*r* = −0.064, *p* = 0.806 > 0.05), nor between the changes in clinical pain scores and the imaging-clinical correlation coefficient (*r* = 0.079, *p* = 0.763 > 0.05). These results indicated that the magnitude of clinical pain relief had no obvious linear association with the changes in neuroimaging indicators or the strength of the imaging-clinical association.

**Table 2:** Results of Spearman's rank correlation analysis among imaging index changes, clinical pain changes, and imaging-clinical correlation coefficients

Association	Spearman's *r*	*P*-value	95% Confidence interval
Imaging index change vs Imaging-clinical correlation *r*	0.931	5.74 × 10^-^⁸	0.812–0.978
Imaging index change vs. Clinical pain change	–0.064	0.806	–0.468 – 0.357
Clinical pain change vs. Imaging-clinical correlation *r*	0.079	0.763	–0.449 – 0.374

"Imaging Index Change" refers to the magnitude of changes in central sensitization-related neuroimaging indicators after acupuncture intervention. "Clinical Pain Change" denotes the degree of reduction in clinical pain scores (VAS/NRS or equivalent) following intervention. "Imaging-Clinical Correlation r" represents the correlation coefficient between neuroimaging indicators and clinical pain outcomes in individual included studies. VAS = Visual Analogue Scale; NRS = Numerical Rating Scale.

### 3.5 Subgroup analysis

#### 3.5.1 Subgroup of pain subtypes

For the osteoarticular pain subtype, a total of 10 studies (230 cases in the acupuncture group, 193 cases in the control group) were included in the meta-analysis (Supplementary Figure S1). Given the high statistical heterogeneity across studies (*I*^2^ = 94%), the random-effects model was applied. The results showed that the pooled mean difference (MD) in the changes of VAS/equivalent pain scores was −3.34 in the acupuncture group compared with the control group (95% CI: −4.86 to −1.81, *Z* = 4.29, *p* < 0.0001), indicating that the magnitude of pain relief in the acupuncture group was significantly greater than that in the control group. The high heterogeneity within this subgroup was mainly attributed to the variations in sample size, pain assessment scales and control intervention types across the included studies.

For the migraine subtype, 3 studies (44 cases in the acupuncture group, 44 cases in the control group) were included (Supplementary Figure S2). The random-effects model was adopted due to high heterogeneity (*I*^2^ = 90%). The pooled MD in the changes of VAS pain scores was −2.41 for the acupuncture group versus the control group (95% CI: −4.84 to 0.02, *Z* = 1.94, *p* = 0.05), suggesting a trend toward superior pain relief in the acupuncture group, albeit without a statistically significant difference.

#### 3.5.2 Subgroup stratified by control intervention type

Subgroup analyses were performed by stratifying the included studies into three categories according to the control intervention type: sham acupuncture, conventional therapy, and blank control. Thirteen studies were included in the sham acupuncture subgroup, and pooled analysis (Supplementary Figure S3) demonstrated that verum acupuncture significantly reduced the VAS scores of patients with chronic pain compared with sham acupuncture (pooled mean difference MD = −0.90, 95% CI: −1.15 to −0.65, *p* < 0.00001). Heterogeneity testing indicated high statistical heterogeneity across the included studies (*I*^2^ = 72%, *p* < 0.0001), which was speculated to be associated with variations in pain subtypes and acupuncture protocols.

Two studies were included in the conventional therapy subgroup (Supplementary Figure S4). Pooled analysis revealed no significant difference in pain relief between verum acupuncture and conventional therapy (pooled mean difference MD = 2.93, 95% CI: −24.61 to 30.47, *p* = 0.83). Extremely high heterogeneity was observed among these studies (*I*^2^ = 97%, *p* < 0.00001), which was presumed to stem from differences in pain subtypes and efficacy assessment indicators.

Four studies were included in the blank control (waitlist) subgroup. Pooled analysis (Supplementary Figure S5) showed that verum acupuncture led to a significant reduction in VAS scores of chronic pain patients compared with blank control (pooled mean difference MD = −2.05, 95% CI: −2.42 to −1.68, *p* < 0.00001). Heterogeneity testing suggested extremely high statistical heterogeneity across the studies (*I*^2^ = 94%, *p* < 0.00001), which was hypothesized to be related to variations in pain subtypes and sample sizes among the included studies.

### 3.6 Sensitivity analysis and publication bias

#### 3.6.1 Sensitivity analysis

To address the high heterogeneity observed in the changes of VAS scores, a one-study-at-a-time approach was adopted to perform the sensitivity analysis in this study. The results (Supplementary Table S2) showed that after the sequential exclusion of any individual study, the 95% confidence interval of the pooled mean difference (MD) for VAS score changes did not cross the null line (0), with all corresponding *p*-values less than 0.00001. Specifically, the exclusion of Jun Zhou (2023), a study with a relatively high statistical weight, resulted in a further enhanced effect size with a pooled MD of −2.89 (95% CI: −3.76 to −2.02). For the exclusion of the remaining studies, the pooled MD values ranged from −2.15 to −2.40, and the overall direction of the effect was consistent with that of the original pooled results. These findings indicated that the pooled effect of VAS score changes in this study had good stability and was not influenced by any single study.

#### 3.6.2 Publication bias

The results of publication bias detection for neuroimaging indicators associated with altered central pain processing were as follows: in the funnel plot for the ACC + insula indicators (Supplementary Figure S6), the data points of the 8 included studies were evenly distributed on both sides of the null line, presenting an essentially symmetric pattern; the funnel plot for the S1 + thalamus indicators (Supplementary Figure S7) also showed good symmetry with no obvious deviation of data points. Although a slight unevenness in data point distribution was observed in the funnel plot for the DMN network indicators (Supplementary Figure S8), no significant asymmetric trend was identified when considering the small number of included studies (*n* = 7). Further Egger’s test and Begg’s test were conducted, with the *t*-values of Egger’s test for the ACC + insula, S1 + thalamus and DMN network indicators being 1.21 (*p* = 0.26), 1.07 (*p* = 0.31) and 1.53 (*p* = 0.17), respectively, and the *Z*-values of Begg’s test being 0.89 (*p* = 0.37), 0.74 (*p* = 0.46), and 1.12 (*p* = 0.26), respectively. The *p*-values of all aforementioned tests were greater than 0.05, indicating that there was no significant publication bias in the pooled results of neuroimaging indicators related to altered central pain processing in this study.

For the changes in VAS scores, the funnel plot (Supplementary Figure S9) showed that the data points of the 17 included studies were generally distributed on both sides of the null line; a slight deviation of individual data points was noted, yet no obvious asymmetric trend was observed. Further Egger’s test and Begg’s test were performed, yielding a *t*-value of 1.87 (*p* = 0.08) for Egger’s test and a *Z*-value of 1.24 (*p* = 0.21) for Begg’s test, which indicated the absence of significant publication bias in the pooled results of VAS score changes. For the pain relief rate, the funnel plot (Supplementary Figure S10) exhibited an even distribution of data points within the confidence interval of the plot with a well-symmetric pattern; Egger’s test produced a *t*-value of 0.94 (*p* = 0.36) and Begg’s test a *Z*-value of 0.67 (*p* = 0.50), which further confirmed that there was no significant publication bias in the pooled results of the pain relief rate.

### 3.7 GRADE assessment of evidence quality

The GRADE system was applied to rate the quality of evidence for six core outcome measures in this study. The results (Supplementary Table S3) showed that the quality of evidence for the pain relief rate (defined as a reduction of ≥2 points in VAS/NRS scores) was rated as high, with no downgrading factors identified, good consistency across study results and a clear clinical implication. The quality of evidence for the improvement of ACC + insula neuroimaging indicators, improvement of S1 + thalamus neuroimaging indicators, reduction in VAS scores, and incidence of adverse events was all rated as moderate. The primary downgrading factors were inadequate reporting of randomization and allocation concealment in some studies, or incomplete data on adverse events. The quality of evidence for the improvement of DMN network neuroimaging indicators was rated as low, owing to some concerns regarding the risk of bias in partial studies and the insufficient explanation for the high statistical heterogeneity (*I*^2^ = 76%) across included studies. Overall, the quality of evidence for the core clinical outcomes (pain relief rate and VAS score reduction) and safety indicators was deemed to be relatively reliable.

## 4 Discussion

This systematic review and meta-analysis integrated neuroimaging and clinical evidence from 17 high-quality randomized controlled trials involving 750 patients. The findings reveal the central mechanisms and clinical value of acupuncture in treating chronic pain associated with altered central pain processing. The results confirmed that acupuncture exerts a dual effect. It not only alleviates clinical pain symptoms but also modulates abnormal brain network function related to altered central pain processing. These findings provide objective neurobiological evidence for the clinical application of acupuncture in chronic pain management.

### 4.1 Modulatory effects on brain networks associated with altered central pain processing

A core characteristic of altered central pain processing is the abnormally elevated excitability of pain processing pathways. It is linked to multidimensional functional impairments including pain emotion and perception, sensory information integration, and cognitive regulation (34). The findings of this study demonstrate that the therapeutic effects of acupuncture are achieved through the targeted modulation of three core brain networks. This finding is highly consistent with the neuroplasticity theory of chronic pain.

In the dimension of emotional perception, the ACC and insula serve as key hubs for encoding pain-related negative emotions and integrating somatosensory signals (35). In the state of chronic pain, these brain regions exhibit excessive activation, which amplifies the emotional burden of pain and exacerbates pain persistence. The present study confirmed that acupuncture can significantly restore the functional indicators of the ACC and insula network. This finding is consistent with previous neuroimaging studies showing that acupuncture alleviates pain-related anxiety and distress by inhibiting abnormal activation of the ACC (36). This suggests that acupuncture can regulate the emotional valence of pain and break the cycle of pain amplification driven by emotional distress, thereby laying a foundation for achieving long-term analgesia.

In the sensory integration pathway, the S1 and thalamus form the core neural circuit for the transmission and processing of nociceptive signals (7, 37). Under the state of altered central pain processing, the elevated excitability of this pathway leads to abnormal manifestations such as reduced pain threshold and allodynia (38). The present study found that acupuncture can enhance the sensory integration function of the S1 and thalamus network. This effect may be associated with inhibition of pronociceptive mediator release, such as substance P in the spinal dorsal horn, as well as regulation of opioid receptor expression (39). This extends the analgesic mechanism of acupuncture from the peripheral level to the central level, enabling multi-level regulation of pain signal transmission. This aligns with the neuromodulatory mechanism of spinal cord stimulation (SCS), which inhibits nociceptive signal transmission by regulating GABAergic interneuron activity and reducing the excitability of wide dynamic range (WDR) neurons in the spinal dorsal horn (40).

The DMN, centered on the PCC, is involved in the processes of self-referential processing and attention allocation (9). Patients with chronic pain often exhibit abnormal functional connectivity of the DMN (41), which results in excessive attention to pain signals and impairs daily functioning. Despite high heterogeneity across studies, the results confirmed that acupuncture can correct DMN dysfunction. This effect may be related to differences in pain subtypes, such as varying degrees of DMN impairment between osteoarticular pain and migraine, as well as variations in acupuncture treatment courses (42). Trends from subgroup analyses suggest that long-course acupuncture may exert a more persistent modulatory effect on the DMN.

### 4.2 Implications for evidence-based practice

This study confirmed that acupuncture can significantly reduce pain scores and improve the pain relief rate in patients with chronic pain. The magnitude of pain reduction exceeded the minimal clinically important difference of 1.5 points on the Visual Analogue Scale, indicating definite clinical significance. The pain relief rate in the acupuncture group was 4.3 times that in the control group, with no significant heterogeneity across studies, reflecting a stable therapeutic effect. This can be attributed to the adoption of standardized acupuncture protocols and sham acupuncture-controlled designs in most included studies, which reduced the variability in intervention implementation.

Notably, a high degree of heterogeneity was observed in the analysis of pain score changes, which may stem from several sources. First, differences in pain subtypes may lead to divergent acupuncture responses; osteoarticular pain is mainly characterized by altered central pain processing induced by peripheral tissue injury, while migraine involves neurovascular dysfunction and neurotransmitter imbalance. Second, variations in outcome measures may also affect the pooled effect size results; although the VAS, Numerical Rating Scale (NRS), and McGill Pain Questionnaire (MPQ) are all validated pain scales, their differing sensitivities and scoring criteria may lead to variations in the pooled effect size. Third, diversity in acupuncture modalities also contributes to heterogeneity; manual acupuncture focuses on the central regulatory effect induced by deqi, whereas electroacupuncture is more likely to activate the spinal descending analgesic pathway, and these two modalities differ in underlying mechanisms. Fourth, differences in control types also contribute to heterogeneity; sham acupuncture can effectively exclude non-specific placebo effects, whereas waitlist control may overestimate therapeutic efficacy due to patient expectancy bias.

Safety analysis revealed that acupuncture-related adverse events were predominantly mild local reactions, with an incidence rate ranging from 3.9 to 10.0%, and no serious adverse events were reported. This finding is consistent with previous systematic reviews concluding that acupuncture has a significantly better safety profile than opioid analgesics and nonsteroidal anti-inflammatory drugs. Therefore, acupuncture is suitable for long-term application. However, 11 studies did not report adverse events in detail, which may indicate the presence of reporting bias. Future studies need to strengthen the standardized monitoring and documentation of adverse events.

### 4.3 Subgroup analyses: a precision medicine perspective

Subgroup analyses provide important insights for the individualized treatment of acupuncture in chronic pain. For osteoarticular pain, acupuncture exerted a more prominent analgesic effect. This may be associated with its regulatory role in peripheral nociceptive pathways, which are affected by both joint inflammation and altered central pain processing. In contrast, acupuncture showed a positive trend in efficacy for migraine treatment without reaching statistical significance. This could be attributed to the small sample size of only three studies and high heterogeneity arising from variations in migraine attack frequency, disease duration, and comorbidities such as anxiety and sleep disorders. Larger-scale randomized controlled trials are warranted to verify the efficacy of acupuncture for migraine and optimize treatment protocols such as acupoint selection and stimulation parameters.

In the subgroup stratified by control intervention type, acupuncture was superior to sham acupuncture and blank control, confirming that its analgesic effect extends beyond the placebo effect. However, no significant difference was observed between acupuncture and conventional therapy. This may be related to the small sample size in the conventional therapy subgroup, which included only two studies, and the high heterogeneity of conventional treatment modalities such as physical therapy and oral analgesics. Future head-to-head trials that use standardized conventional therapy as the control are needed to clarify the relative efficacy of acupuncture.

Regarding intervention parameters, most included studies emphasized deqi induction, with a needle retention time of 20 to 30 min and a treatment course of 4 to 8 weeks. Deqi is characterized by sensations of soreness, numbness, and distension at acupoints. It is recognized as a key determinant of acupuncture efficacy because it can activate peripheral nerve endings and enhance central regulatory effects. Appropriate extension of the treatment course may consolidate the neuroplastic changes in pain-related brain networks, highlighting the importance of standardized parameters for stabilizing clinical outcomes.

### 4.4 Limitations of the study

Despite these strengths, the study also has certain limitations. First, the number of included studies is relatively small at seventeen, and the sample size of some subgroups such as the conventional therapy control subgroup is limited, which may compromise the reliability of the relevant results. Second, the diversity of neuroimaging indicators, such as fractional amplitude of low-frequency fluctuation, regional homogeneity, and resting-state functional connectivity, has prevented the pooling of partial data. This limits the direct comparison of different analytical methods. Third, the follow-up period of most studies is relatively short, ranging from 4–8 weeks. This makes it impossible to evaluate the long-term regulatory effect of acupuncture on altered central pain processing and its impact on pain recurrence. Fourth, some studies failed to report detailed acupuncture protocols such as specific acupoint combinations and stimulation intensity, which may affect the reproducibility of the intervention. Fifth, although funnel plots, Egger’s tests and Begg’s tests did not indicate significant publication bias, the potential for underlying publication bias cannot be completely ruled out.

### 4.5 Future research directions

Based on the findings and limitations of this study, future research can focus on the following directions. First, large-scale, multicenter, double-blind randomized controlled trials with sham acupuncture as control should be conducted to verify the efficacy of acupuncture for specific pain subtypes such as migraine and fibromyalgia. These studies can also optimize the corresponding treatment protocols. Second, standardized guidelines for acupuncture interventions should be developed. These guidelines should specify acupoint selection, stimulation parameters including electroacupuncture frequency and intensity, and treatment courses to reduce heterogeneity across studies. Third, multimodal neuroimaging techniques, such as functional magnetic resonance imaging combined with positron emission tomography or proton magnetic resonance spectroscopy, should be adopted. These techniques can explore the effects of acupuncture on cerebral metabolism and neurotransmitter concentrations, including gamma-aminobutyric acid and glutamate, thereby further elucidating the underlying central mechanisms. Fourth, longitudinal studies should be carried out to track the dynamic changes in altered central pain processing-related brain networks during the course of acupuncture treatment. These studies will provide evidence for individualized clinical treatment. Fifth, explore the synergistic effects of acupuncture combined with other interventions such as opioid analgesics and physical therapy, to optimize comprehensive treatment strategies for chronic pain.

### 4.6 Implications for clinical practice

This study offers important implications for clinical practice. First, acupuncture can be used as a preferred non-pharmacological intervention for chronic pain, particularly in selected populations such as patients with osteoarticular pain and those who cannot tolerate conventional analgesics. Second, clinical application should attach great importance to deqi induction, with a recommended needle retention time of 20 to 30 min and a treatment course of no less than 4 weeks, to ensure a sufficient central regulatory effect. Third, individualized protocols need to be formulated according to different pain subtypes. For example, local acupoints combined with distal acupoints such as Zusanli and Hegu can be selected for osteoarticular pain, while acupoints regulating the meridians of the head and neck such as Fengchi and Taiyang can be prioritized for migraine. Fourth, for patients with poor response to opioid analgesics or those who are concerned about adverse reactions, acupuncture can serve as a safe alternative. This approach supports the opioid tapering strategy in the management of chronic pain.

## Data Availability

The original contributions presented in the study are included in the article/Supplementary material, further inquiries can be directed to the corresponding author/s.
