# Supplementary material for: A meta-analysis of neuroimaging evidence for acupuncture-mediated modulation of altered central pain processing in patients with chronic pain
Source: Front Neurol. 2026 May 1;17:1809628. doi: 10.3389/fneur.2026.1809628 (PMC13177863; doi:10.3389/fneur.2026.1809628)
Supplement: Supplementary file 1 [file Supplementary_file_1.docx]

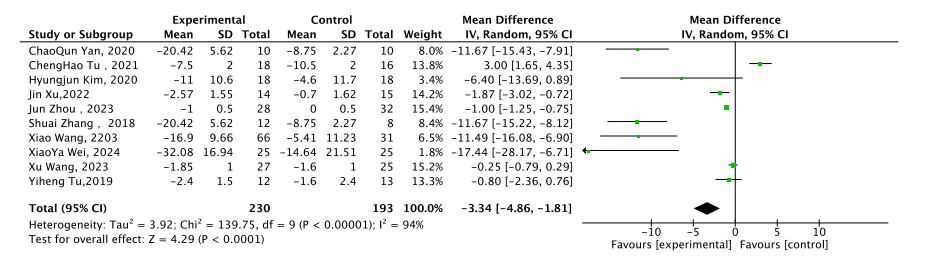


**Figure S1 Forest plot of VAS/equivalent pain score changes in the osteoarticular pain subtype subgroup**

MD = Mean Difference; CI = Confidence Interval; VAS = Visual Analogue Scale


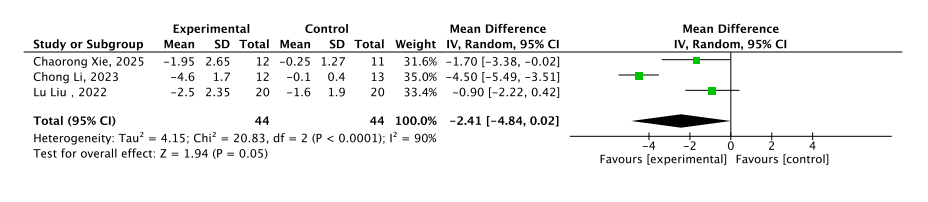


Figure S2 Forest plot of VAS pain score changes in the migraine subtype subgroup

MD = Mean Difference; CI = Confidence Interval; VAS = Visual Analogue Scale


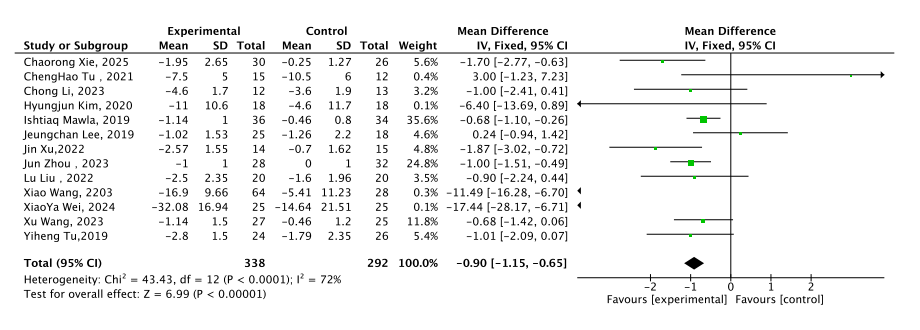


Figure S3 Forest plot of VAS score changes in the sham acupuncture control subgroup

MD = Mean Difference; CI = Confidence Interval; VAS = Visual Analogue Scale


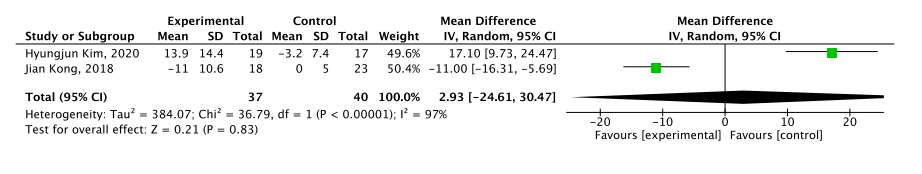


Figure S4 Forest plot of VAS score changes in the conventional therapy control subgroup

MD = Mean Difference; CI = Confidence Interval; VAS = Visual Analogue Scale


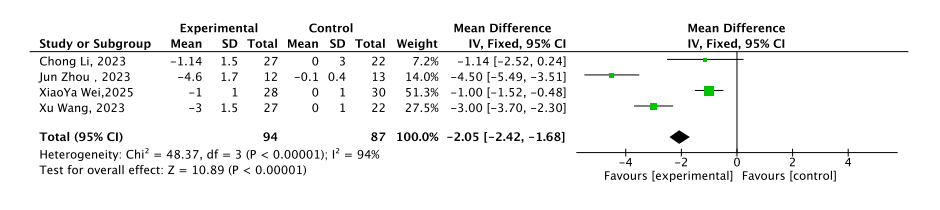


Figure S5 Forest plot of VAS score changes in the blank control (waitlist) subgroup

MD = Mean Difference; CI = Confidence Interval; VAS = Visual Analogue Scale


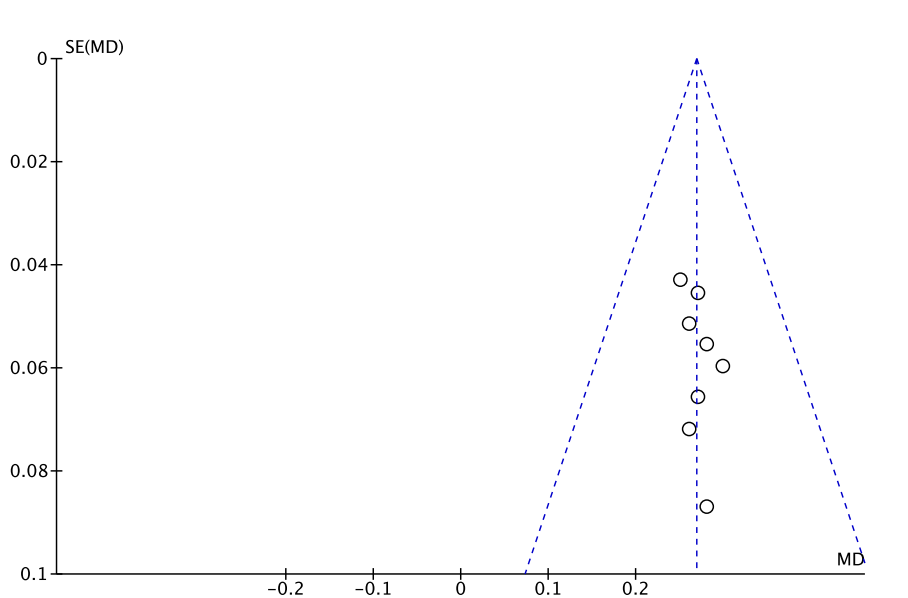


Figure S6 Funnel plot for publication bias detection of ACC + insula-related neuroimaging indicators

SE(MD) = Standard Error of Mean Difference; MD = Mean Difference; ACC = Anterior Cingulate Cortex


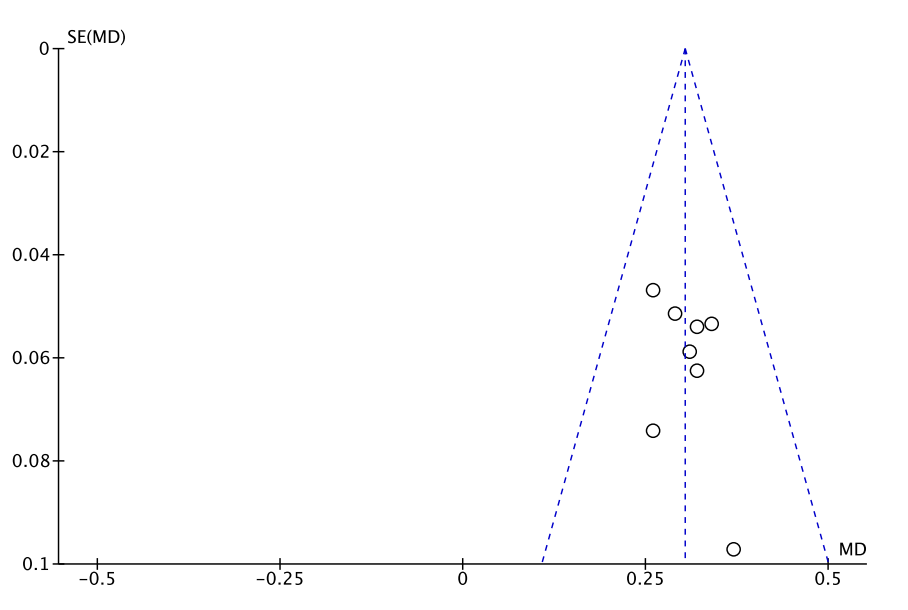


Figure S7 Funnel plot for publication bias detection of S1 + thalamus-related neuroimaging indicators

SE(MD) = Standard Error of Mean Difference; MD = Mean Difference; S1 = Primary Somatosensory Cortex


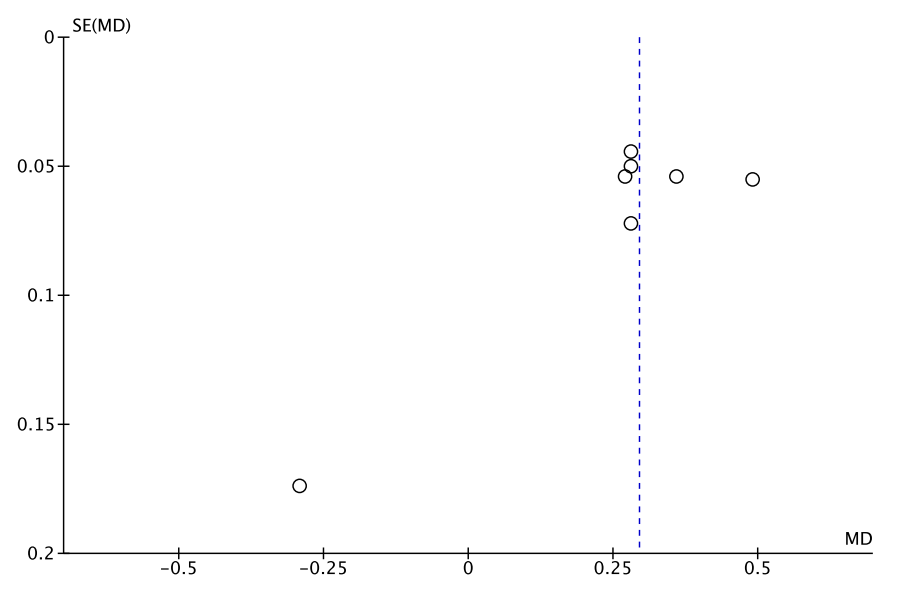


Figure S8 Funnel plot for publication bias detection of DMN network-related neuroimaging indicators

SE(MD) = Standard Error of Mean Difference; MD = Mean Difference; DMN = Default Mode Network


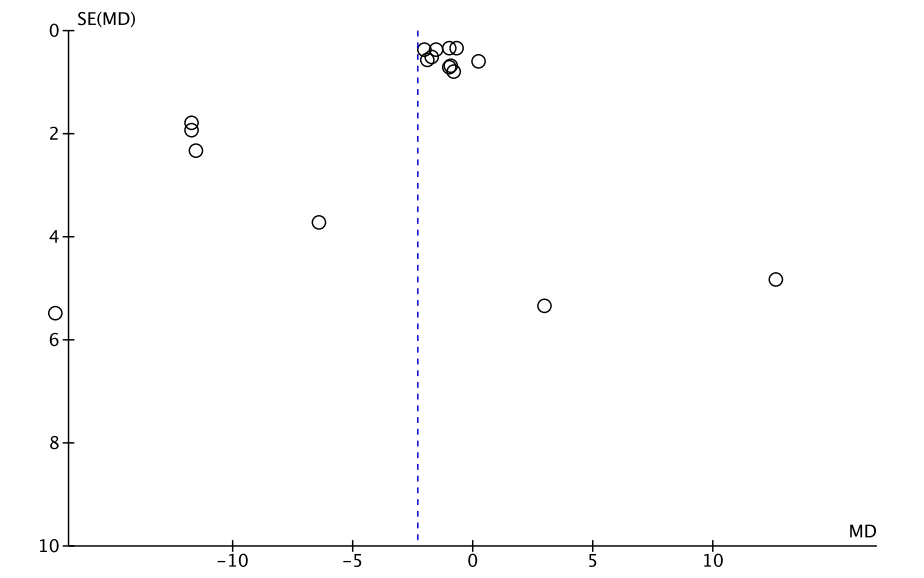


Figure S9 Funnel plot for publication bias detection of VAS score changes

SE(MD) = Standard Error of Mean Difference; MD = Mean Difference; VAS = Visual Analogue Scale


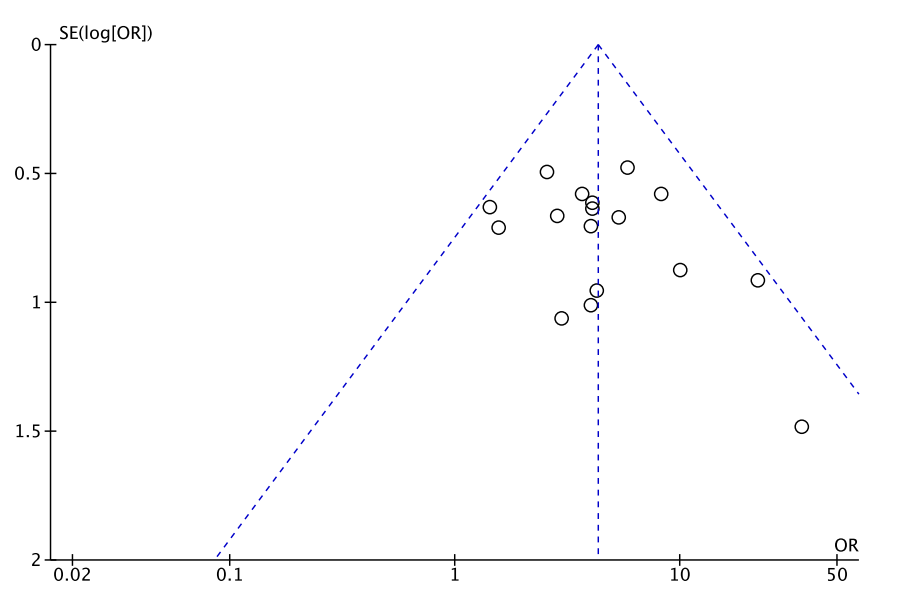


Figure S10 Funnel plot for publication bias detection of pain relief rate

SE(log(OR)) = Standard Error of log-transformed Odds Ratio; OR = Odds Ratio
