# Supplementary material for: A meta-analysis of neuroimaging evidence for acupuncture-mediated modulation of altered central pain processing in patients with chronic pain
Source: Front Neurol. 2026 May 1;17:1809628. doi: 10.3389/fneur.2026.1809628 (PMC13177863; doi:10.3389/fneur.2026.1809628)
Supplement: Supplementary file 2 [file Supplementary_file_2.docx]

| **Section and Topic** | **Item #** | **Checklist item** | **Location where item is reported** |
| --- | --- | --- | --- |
| **TITLE** | | |  |
| Title | 1 | Identify the report as a systematic review. | Title (implied in manuscript core content; abstract and methods explicitly state "systematic review and meta-analysis") |
| **ABSTRACT** | | |  |
| Abstract | 2 | See the PRISMA 2020 for Abstracts checklist. | Abstract (includes objective, methods, results, conclusion consistent with PRISMA 2020 Abstract criteria) |
| **INTRODUCTION** | | |  |
| Rationale | 3 | Describe the rationale for the review in the context of existing knowledge. | 1 Introduction (1st-3rd paragraphs: chronic pain burden, central sensitization as core mechanism, acupuncture clinical application and unclear central regulatory mechanisms) |
| Objectives | 4 | Provide an explicit statement of the objective(s) or question(s) the review addresses. | 1 Introduction (4th paragraph) & Abstract (Objective section) |
| **METHODS** | | |  |
| Eligibility criteria | 5 | Specify the inclusion and exclusion criteria for the review and how studies were grouped for the syntheses. | 2.1 Inclusion and Exclusion Criteria (PICO framework for participants, interventions, comparators, outcomes, study design; subgroup stratification for syntheses stated in 2.5 Statistical Analysis Methods) |
| Information sources | 6 | Specify all databases, registers, websites, organisations, reference lists and other sources searched or consulted to identify studies. Specify the date when each source was last searched or consulted. | 2.2 Literature Search Strategy (English: PubMed, EMBASE, Cochrane Library, Web of Science, Scopus; Chinese: CNKI, Wanfang, VIP, CBM; grey literature: ClinicalTrials.gov, Chinese Clinical Trial Registry, conference proceedings, dissertations; search timeframe: 2016.01.01–2025.12.31) |
| Search strategy | 7 | Present the full search strategies for all databases, registers and websites, including any filters and limits used. | 2.2 Literature Search Strategy (combined subject headings + free text terms; core search terms categorized by intervention, disease, neuroimaging, study design; time filter 2016–2025) |
| Selection process | 8 | Specify the methods used to decide whether a study met the inclusion criteria of the review, including how many reviewers screened each record and each report retrieved, whether they worked independently, and if applicable, details of automation tools used in the process. | 2.3 Study Selection and Data Extraction (2 independent researchers; EndNote X9 for automatic de-duplication; third researcher for consensus on discrepancies; target discrepancy rate ≤5%) |
| Data collection process | 9 | Specify the methods used to collect data from reports, including how many reviewers collected data from each report, whether they worked independently, any processes for obtaining or confirming data from study investigators, and if applicable, details of automation tools used in the process. | 2.3 Study Selection and Data Extraction (2 independent researchers; Excel 2021 standardized extraction form; cross-check for consistency; no investigator data confirmation stated) |
| Data items | 10a | List and define all outcomes for which data were sought. Specify whether all results that were compatible with each outcome domain in each study were sought (e.g. for all measures, time points, analyses), and if not, the methods used to decide which results to collect. | 2.1 Inclusion and Exclusion Criteria (Outcomes section: core = neuroimaging indices; secondary = clinical/safety indices; associative = imaging-clinical correlation coefficient; all compatible results for each domain sought) |
|  | 10b | List and define all other variables for which data were sought (e.g. participant and intervention characteristics, funding sources). Describe any assumptions made about any missing or unclear information. | 2.3 Study Selection and Data Extraction (extracted: basic study characteristics, participant demographics, intervention/control details, risk of bias information; no assumptions about missing/unclear information stated) |
| Study risk of bias assessment | 11 | Specify the methods used to assess risk of bias in the included studies, including details of the tool(s) used, how many reviewers assessed each study and whether they worked independently, and if applicable, details of automation tools used in the process. | 2.4 Risk of Bias Assessment (Cochrane RoB 2.0; 5 core domains; 3 rating tiers; 2 independent researchers; third researcher for consensus; no automation tools) |
| Effect measures | 12 | Specify for each outcome the effect measure(s) (e.g. risk ratio, mean difference) used in the synthesis or presentation of results. | 2.5 Statistical Analysis Methods (continuous variables: MD/SMD; dichotomous variables: OR; all with 95% CI) |
| Synthesis methods | 13a | Describe the processes used to decide which studies were eligible for each synthesis (e.g. tabulating the study intervention characteristics and comparing against the planned groups for each synthesis (item #5)). | 2.5 Statistical Analysis Methods (subgroup stratification by pain subtype, control intervention type, acupuncture parameters; consistent with item 5 grouping criteria) |
|  | 13b | Describe any methods required to prepare the data for presentation or synthesis, such as handling of missing summary statistics, or data conversions. | 2.5 Statistical Analysis Methods (no missing summary statistics/data conversion methods stated) |
|  | 13c | Describe any methods used to tabulate or visually display results of individual studies and syntheses. | 2.5 Statistical Analysis Methods (RevMan 5.4 for forest plots/funnel plots; tables for baseline characteristics/results; Figure 1 for study selection flow diagram) |
|  | 13d | Describe any methods used to synthesize results and provide a rationale for the choice(s). If meta-analysis was performed, describe the model(s), method(s) to identify the presence and extent of statistical heterogeneity, and software package(s) used. | 2.5 Statistical Analysis Methods (meta-analysis via RevMan 5.4; heterogeneity: I² + Q test; fixed-effects (I²<50%, P>0.10) vs random-effects (I²≥50%, P≤0.10); rationale: based on heterogeneity magnitude) |
|  | 13e | Describe any methods used to explore possible causes of heterogeneity among study results (e.g. subgroup analysis, meta-regression). | 2.5 Statistical Analysis Methods (subgroup analysis by pain subtype, control intervention type, acupuncture parameters; no meta-regression) |
|  | 13f | Describe any sensitivity analyses conducted to assess robustness of the synthesized results. | 2.5 Statistical Analysis Methods (one-study-at-a-time sensitivity analysis for VAS score changes) |
| Reporting bias assessment | 14 | Describe any methods used to assess risk of bias due to missing results in a synthesis (arising from reporting biases). | 3.6 Publication Bias (funnel plot analysis; Egger’s test; Begg’s test; trim-and-fill method for correction if bias identified) |
| Certainty assessment | 15 | Describe any methods used to assess certainty (or confidence) in the body of evidence for an outcome. | 2.5 Statistical Analysis Methods (GRADE approach; 5 evaluation domains; 4 evidence quality levels: high/moderate/low/very low) |
| **RESULTS** | | |  |
| Study selection | 16a | Describe the results of the search and selection process, from the number of records identified in the search to the number of studies included in the review, ideally using a flow diagram. | 3.1 Study Identification and Selection Outcomes (505 records identified → 52 duplicates removed → 453 title/abstract screening → 355 full-text retrieval → 335 full-text assessment → 17 studies included; Figure 1 flow diagram) |
|  | 16b | Cite studies that might appear to meet the inclusion criteria, but which were excluded, and explain why they were excluded. | 3.1 Study Identification and Selection Outcomes (318 studies excluded: 203 non-RCTs, 52 ineligible interventions, 63 incomplete outcome measures; no individual excluded study citation) |
| Study characteristics | 17 | Cite each included study and present its characteristics. | 3.2 Baseline Characteristics of Included Studies (17 studies cited [16–32]; Table 1 for baseline characteristics: pain subtype, sample size, intervention details, neuroimaging/clinical outcomes) |
| Risk of bias in studies | 18 | Present assessments of risk of bias for each included study. | 3.3 Risk of Bias Assessment Results (Figure 2 for risk of bias assessment; 13 studies low risk, 4 studies some concerns; no high risk bias; domain-specific bias results described) |
| Results of individual studies | 19 | For all outcomes, present, for each study: (a) summary statistics for each group (where appropriate) and (b) an effect estimate and its precision (e.g. confidence/credible interval), ideally using structured tables or plots. | 3.4 Results of Meta-Analysis (forest plots for neuroimaging/clinical outcomes (Figures 3–7); summary statistics/effect estimates with 95% CI for each outcome; subgroup analysis figures (S1–S5)) |
| Results of syntheses | 20a | For each synthesis, briefly summarise the characteristics and risk of bias among contributing studies. | 3.4 Results of Meta-Analysis (neuroimaging/clinical syntheses: sample size of acupuncture/control groups, risk of bias level (low/some concerns) summarised for each synthesis) |
|  | 20b | Present results of all statistical syntheses conducted. If meta-analysis was done, present for each the summary estimate and its precision (e.g. confidence/credible interval) and measures of statistical heterogeneity. If comparing groups, describe the direction of the effect. | 3.4 Results of Meta-Analysis (ACC+insula: MD=0.27, 95%CI=0.23–0.31, I²=0%; S1+thalamus: MD=0.30,95%CI=0.26–0.34,I²=0%; DMN: MD=0.29,95%CI=0.20–0.39,I²=76%; VAS: MD=-2.31,95%CI=-3.27–-1.36,I²=86%; pain relief rate: OR=4.30,95%CI=3.14–5.90,I²=0%; all acupuncture group effects in favorable direction) |
|  | 20c | Present results of all investigations of possible causes of heterogeneity among study results. | 3.5 Subgroup analysis & 3.4.2 Clinical Outcome Measures (heterogeneity causes speculated: pain subtype, outcome measures, acupuncture modalities, control types; subgroup analysis results for each stratification factor presented) |
|  | 20d | Present results of all sensitivity analyses conducted to assess the robustness of the synthesized results. | 3.6.1 Sensitivity Analysis (Table S2; one-study-at-a-time for VAS; pooled MD 95%CI no null line crossing; P<0.00001; effect direction consistent; Jun Zhou (2023) exclusion enhanced effect size) |
| Reporting biases | 21 | Present assessments of risk of bias due to missing results (arising from reporting biases) for each synthesis assessed. | 3.6 Publication Bias (funnel plots S6–S10; Egger’s/Begg’s test P-values >0.05 for all syntheses; no significant publication bias for neuroimaging/VAS/pain relief rate) |
| Certainty of evidence | 22 | Present assessments of certainty (or confidence) in the body of evidence for each outcome assessed. | 3.7 GRADE Assessment of Evidence Quality (Table S3; pain relief rate: high; ACC+insula/S1+thalamus/VAS/adverse events: moderate; DMN: low; downgrading factors described) |
| **DISCUSSION** | | |  |
| Discussion | 23a | Provide a general interpretation of the results in the context of other evidence. | 4 Discussion (4.1 Modulatory Effects on Brain Networks; links results to chronic pain neuroplasticity theory, previous acupuncture/neuroimaging studies) |
|  | 23b | Discuss any limitations of the evidence included in the review. | 4.4 Limitations of the Study (small number of included studies, small subgroup sample sizes, diverse neuroimaging indicators, short follow-up, incomplete acupuncture protocol reporting) |
|  | 23c | Discuss any limitations of the review processes used. | 4.4 Limitations of the Study (potential reporting bias for adverse events, inability to rule out underlying publication bias, no meta-regression for heterogeneity) |
|  | 23d | Discuss implications of the results for practice, policy, and future research. | 4.5 Future Research Directions & 4.6 Implications for Clinical Practice (clinical practice: acupuncture as first-line non-pharmacological intervention, standardized parameters; future research: large multicenter RCTs, multimodal neuroimaging, longitudinal studies; no policy implications stated) |
| **OTHER INFORMATION** | | |  |
| Registration and protocol | 24a | Provide registration information for the review, including register name and registration number, or state that the review was not registered. | 2 Methods (1st paragraph; PROSPERO, CRD420261290299) |
|  | 24b | Indicate where the review protocol can be accessed, or state that a protocol was not prepared. | 2 Methods (1st paragraph; protocol registered in PROSPERO; no specific access link stated) |
|  | 24c | Describe and explain any amendments to information provided at registration or in the protocol. | No relevant content (no amendments to registered protocol stated) |
| Support | 25 | Describe sources of financial or non-financial support for the review, and the role of the funders or sponsors in the review. | Declarations (Added funding information; Natural Science Foundation of Shandong Province, ZR2025MS1504; no funder role stated) |
| Competing interests | 26 | Declare any competing interests of review authors. | Declarations (Competing Interests; authors declare no competing interests) |
| Availability of data, code and other materials | 27 | Report which of the following are publicly available and where they can be found: template data collection forms; data extracted from included studies; data used for all analyses; analytic code; any other materials used in the review. | Data Availability (data from 17 included RCTs: public via journal repositories/research platforms; analytic code (RevMan 5.4): available from corresponding author on reasonable request; no template forms/extracted data public access stated) |

*From:*  Page MJ, McKenzie JE, Bossuyt PM, Boutron I, Hoffmann TC, Mulrow CD, et al. The PRISMA 2020 statement: an updated guideline for reporting systematic reviews. BMJ 2021;372:n71. doi: 10.1136/bmj.n71. This work is licensed under CC BY 4.0. To view a copy of this license, visit <https://creativecommons.org/licenses/by/4.0/>
