# Supplementary material for: A meta-analysis of neuroimaging evidence for acupuncture-mediated modulation of altered central pain processing in patients with chronic pain
Source: Front Neurol. 2026 May 1;17:1809628. doi: 10.3389/fneur.2026.1809628 (PMC13177863; doi:10.3389/fneur.2026.1809628)
Supplement: Supplementary file 3 [file Table_1.docx]

Table S1 Adverse events reported in the included studies

| Study ID | Author, Year | Pain Subtype | Adverse Events in Experimental Group (Acupuncture) | Adverse Events in Control Group (Sham Acupuncture / Other Controls) |
| --- | --- | --- | --- | --- |
| 1 | XiaoYa Wei, 2024 | Chronic pain (chronic sciatica) | 3 cases of mild ecchymosis around acupuncture points | No adverse events reported |
| 2 | Xu Wang, 2023 | Chronic pain (knee osteoarthritis-related pain) | 3 cases (11.1%), including 1 case of subcutaneous ecchymosis and 2 cases of post-acupuncture sensation | 1 case (4%) of subcutaneous ecchymosis |
| 3 | Chong Li, 2023 | Chronic pain (migraine) | No adverse events reported | No adverse events reported |
| 4 | Lu Liu, 2022 | Chronic pain (migraine ) | Not clearly reported | Not clearly reported |
| 5 | Jun Zhou, 2023 | Chronic pain (knee osteoarthritis-related pain) | 4 cases of mild ecchymosis around acupuncture points | 4 cases of mild ecchymosis around acupuncture points; 1 case in the celecoxib group withdrew due to gastric discomfort |
| 6 | Ishtiaq Mawla, 2019 | Chronic pain (fibromyalgia, FM) | Not clearly reported | Not clearly reported |
| 7 | Jian Kong, 2018 | Chronic pain (knee osteoarthritis-related pain) | Not clearly reported | Not clearly reported |
| 8 | Chaorong Xie, 2025 | Chronic pain  (migraine) | 2 cases of mild adverse events (1 case of subcutaneous hemorrhage, 1 case of local pain) | No adverse events reported |
| 9 | Hyungjun Kim, 2020 | Chronic pain  (chronic low back pain, cLBP) | Not clearly reported | Not clearly reported |
| 10 | Xiao Wang, 2023 | Chronic pain  (chronic neck pain, CP) | 4 cases of mild ecchymosis at acupuncture points | 3 cases of mild ecchymosis at acupuncture points |
| 11 | Jin Xu, 2022 | Chronic pain (primary dysmenorrhea, PDM) | Not clearly reported | Not clearly reported |
| 12 | ChaoQun Yan, 2020 | Chronic pain (chronic shoulder pain, CSP) | Not clearly reported | Not clearly reported |
| 13 | XiaoYa Wei, 2025 | Chronic pain (knee osteoarthritis-related pain) | Not clearly reported | Not clearly reported |
| 14 | Jeungchan Lee, 2019 | Chronic pain ( chronic low back pain, cLBP) | Not clearly reported | Not clearly reported |
| 15 | Yiheng Tu, 2019 | Chronic pain (chronic low back pain, cLBP) | Not clearly reported | Not clearly reported |
| 16 | ChengHao Tu, 2021 | Chronic pain (primary dysmenorrhea, PDM) | 1 case took analgesics for non-dysmenorrheal reasons | 1 case took analgesics during menstruation |
| 17 | Shuai Zhang, 2018 | Chronic pain  (chronic shoulder pain, CSP) | Not clearly reported | Not clearly reported |

"Not clearly reported" indicates that the original study did not provide specific information on adverse events.

Adverse events are categorized as mild local reactions unless otherwise specified, with no serious adverse events reported across all included studies.

Abbreviations: cLBP = Chronic Low Back Pain; CSP = Chronic Shoulder Pain; PDM = Primary Dysmenorrhea; FM = Fibromyalgia.
