# Supplementary material for: A meta-analysis of neuroimaging evidence for acupuncture-mediated modulation of altered central pain processing in patients with chronic pain
Source: Front Neurol. 2026 May 1;17:1809628. doi: 10.3389/fneur.2026.1809628 (PMC13177863; doi:10.3389/fneur.2026.1809628)
Supplement: Supplementary file 4 [file Table_2.docx]

Table S2 Sensitivity analysis results for changes in VAS scores

| Excluded Study | Pooled MD (95% CI) | P-value | Stability Conclusion |
| --- | --- | --- | --- |
| Original (no exclusion) | -2.31 (-3.27 to -1.36) | <0.00001 | Significant |
| ChaoQun Yan, 2020 | -2.25 (-3.18 to -1.32) | <0.00001 | Significant |
| Chaorong Xie, 2025 | -2.40 (-3.35 to -1.45) | <0.00001 | Significant |
| ChengHao Tu, 2021 | -2.35 (-3.30 to -1.40) | <0.00001 | Significant |
| Chong Li, 2023 | -2.28 (-3.22 to -1.34 | <0.00001 | Significant |
| Hyungjun Kim, 2020 | -2.33 (-3.28 to -1.38) | <0.00001 | Significant |
| Ishtiaq Mawla, 2019 | -2.37 (-3.32 to -1.42) | <0.00001 | Significant |
| Jeungchan Lee, 2019 | -2.30 (-3.25 to -1.35) | <0.00001 | Significant |
| Jian Kong, 2018 | -2.32 (-3.27 to -1.37) | <0.00001 | Significant |
| Jin Xu, 2022 | -2.35 (-3.30 to -1.40) | <0.00001 | Significant |
| Jun Zhou, 2023 | -2.89 (-3.76 to -2.02) | <0.00001 | Significant |
| Lu Liu, 2022 | -2.33 (-3.28 to -1.38) | <0.00001 | Significant |
| Shuai Zhang, 2018 | -2.27 (-3.21 to -1.33) | <0.00001 | Significant |
| Xiao Wang, 2203 | -2.15 (-3.08 to -1.22) | <0.00001 | Significant |
| XiaoYa Wei, 2024 | -2.30 (-3.25 to -1.35) | <0.00001 | Significant |
| XiaoYa Wei, 2025 | -2.34 (-3.29 to -1.39) | <0.00001 | Significant |
| Xu Wang, 2023 | -2.32 (-3.27 to -1.37) | <0.00001 | Significant |
| Yiheng Tu, 2019 | -2.30 (-3.25 to -1.35) | <0.00001 | Significant |

Sensitivity analysis was performed using a one-study-at-a-time approach, sequentially excluding each individual study to assess the stability of the pooled effect size.

"Pooled MD" refers to the pooled mean difference of VAS score changes between the acupuncture group and control group after excluding the corresponding study.

Abbreviations: MD = Mean Difference; CI = Confidence Interval; VAS = Visual Analogue Scale.
