# Supplementary material for: A meta-analysis of neuroimaging evidence for acupuncture-mediated modulation of altered central pain processing in patients with chronic pain
Source: Front Neurol. 2026 May 1;17:1809628. doi: 10.3389/fneur.2026.1809628 (PMC13177863; doi:10.3389/fneur.2026.1809628)
Supplement: Supplementary file 5 [file Table_3.docx]

**Table S3 GRADE evidence quality assessment results for core outcome measures**

| Outcome Measures (Measurement Tool/Follow-up Duration) | Study Type (No. of Studies/Total Sample Size) | Effect Size (95% CI) | Evidence Quality | Downgrading Factors (-1/-2) | Upgrading Factors (+1/+2) | Notes (Clinical Significance) |
| --- | --- | --- | --- | --- | --- | --- |
| Core Outcome 1: Improvement of ACC + Insula Imaging Indices(fMRI-fALFF/ReHo/rsFC; 4-8 weeks post-intervention) | RCT (8 studies/379 participants) | MD=0.27 (0.23~0.31) | Moderate | Risk of bias (-1): Inadequate reporting of randomization/allocation concealment in 5 studies | None | Improved imaging indices indicate regulation of the pain emotion-perception circuit, correlating with clinical analgesic effects |
| Core Outcome 2: Improvement of S1 + Thalamus Imaging Indices(fMRI-rsFC/GMV/FA; 4-8 weeks post-intervention) | RCT (8 studies/387 participants) | MD=0.30 (0.26~0.34) | Moderate | Risk of bias (-1): Same as the above bias issue | None | Enhanced function of the sensory information integration pathway supports the reversal of central sensitization |
| Core Outcome 3: Improvement of DMN Network Imaging IndicesMRI-ReHo/ALFF/rsFC; 4-8 weeks post-intervention) | RCT (7 studies/332 participants) | MD=0.29 (0.20~0.39) | Low | 1. Risk of bias (-1): "Some concerns" regarding bias in some studies; Inconsistency (-1): I²=76%, heterogeneity not fully explained | None | Correction of DMN dysfunction is associated with improved "self-referential pain processing" in chronic pain |
| Core Outcome 4: Reduction in VAS Score/NRS; 4-8 weeks post-intervention) | RCT (17 studies/750 participants) | MD=-2.31 (-3.27~-1.36) | Moderate | Risk of bias (-1): Inadequate reporting of randomization-related information in 5 studies | None | Reduction amplitude >1.5 points (Minimum Clinically Important Difference, MCID), with clear clinical significance |
| Core Outcome 5: Pain Relief RateAS/NRS reduction ≥2 points; 4-8 weeks post-intervention) | RCT (17 studies/750 participants) | OR=4.30 (3.14~5.90) | High | None | None | The probability of pain relief in the acupuncture group is 4.3 times that of the control group, with good result consistency (I²=0%) |
| Core Outcome 6: Adverse Event Rate(Local reactions/serious adverse events; during intervention) | RCT (17 studies/1286 participants) | Incidence local reactions) | Moderate | Imprecision (-1): Incomplete data as 11 studies did not report adverse events | None | No serious adverse events, good safety profile supports clinical application |

Evidence quality grading criteria: High = Very confident that the true effect is close to the estimated value; Moderate = The true effect is likely close to the estimated value, but there is a possibility of difference; Low = Limited confidence, the true effect may be substantially different from the estimated value; Very low = Almost no confidence, the estimated value is likely unreliable.

Downgrading/upgrading basis: Determined with reference to the 5 downgrading factors (risk of bias, inconsistency, indirectness, imprecision, publication bias) and 3 upgrading factors (large effect size, dose-response relationship, robustness to residual confounding) of the GRADE system.

Sample size calculation: The total sample size is the sum of the number of participants in the acupuncture group and the control group, and only the sample size of studies reporting the outcome is counted.

Clinical significance determination: Combined with the minimal clinically important difference (MCID) in the field of chronic pain and the strength of association between neuroimaging indicators and central sensitization.
